# A human erythropoietin-albumin fusion protein tailored for enhanced plasma half-life and delivery across mucosal surfaces

**DOI:** 10.1038/s41467-026-78243-z

**Published:** 2026-10-28

**Authors:** Aina Karen Anthi, Daniela Bejan, Simone Mester, Nijaguna Bethur, Elena Gonzalo-Gil, Sopisa Benjakul, Kristin Hovden Aaen, Tilman Schlothauer, Inger Sandlie, Jan Terje Andersen

**Affiliations:** 1 Precision Immunotherapy Alliance (PRIMA), University of Oslo, Oslo, Norway; 2 Department of Immunology, Oslo University Hospital Rikshospitalet, Oslo, Norway; 3 Department of Pharmacology, Institute of Clinical Medicine, Faculty of Medicine, University of Oslo, Oslo, Norway; 4 The Jackson Laboratory, Bar Harbor, ME USA; 5 The Jackson Laboratory, Alachua, FL USA; 6 Biochemical and Analytical Research, Large Molecule Research, Roche Pharma Research and Early Development (pRED), Roche Innovation Center Munich, Penzberg, Germany; 7 Department of Biosciences, University of Oslo, Oslo, Norway

**Keywords:** Recombinant protein therapy, Drug delivery, Molecular engineering, Protein delivery, Molecular medicine

## Abstract

Erythropoietin (EPO) products stimulate production of erythrocytes in patients with anemia. The drugs are delivered intravenously or subcutaneously up to several times per week due to short plasma half-life. Improving the pharmacokinetic properties of EPO will allow less frequent administration, and thus impact therapeutic utility and patient compliance. In this study, we took advantage of an albumin variant (QMP) engineered for improved engagement of FcRn, a key regulator of the plasma half-life of albumin. After genetic coupling of EPO to QMP, the fusion protein shows favorable neonatal Fc receptor (FcRn) binding, and 5.2-fold prolonged plasma half-life in human FcRn expressing mice upon intravenous administration, when comparing with EPO fused to WT albumin, with a similar half-life obtained following subcutaneous administration. Importantly, both fusions bind the EPO receptor with binding kinetics equal to that of the product darbepoetin alfa. After intravenous administration, mice receiving darbepoetin alfa experiences a faster drop in EPO levels compared to those given EPO-QMP, and moreover, we measure a prolonged increase in erythrocytes with EPO-QMP. Furthermore, intranasal administration results in 5-fold higher circulating amounts of EPO-QMP and a prolonged increase in erythrocytes compared to darbepoetin alfa. Thus, long-acting EPO-QMP could be an attractive design to explore for both invasive and non-invasive delivery to patients with anemia.

## Abstract (web summary)

This study presents a tailored erythropoietin fused to engineered albumin for extended plasma half-life after intravenous and subcutaneous delivery in mice. Following invasive or intranasal delivery, it promotes sustained erythrocyte proliferation.

## Introduction

Recombinant human erythropoietin (EPO) products are widely used in the clinic to boost the production of erythrocytes in patients suffering from anemia due to chronic kidney disease or secondary to other treatments, such as cancer chemotherapy or zidovudine treatment after human immunodeficient virus infection^1–3^. Interestingly, studies have demonstrated that EPO may also offer therapeutic benefit in neurological conditions, like neurodegenerative diseases, as well as for the treatment of schizophrenia and stroke^4,5^. Hence, EPO is used for both short-term and lifelong replacement treatment. The products are generally well-tolerated and frequently administered intravenously or subcutaneously. However, the first marketed recombinant EPO products have a rather short plasma half-life ranging from 4–11 h after intravenous administration in humans, which increases to about a day following subcutaneous administration^6^. To solve this, engineering strategies have been explored to extend the plasma half-life of recombinant EPO. For instance, darbepoetin alfa (Aranesp, Amgen) has five amino acid substitutions that introduce two additional N-glycan sites, compared to the three N- and one O-glycan sites on EPO, extending the plasma half-life in humans from 8.5 to 26.3 h after intravenous administration^2,7,8^. Another example is methoxy polyethylene glycol (PEG)-epoetin beta (Mircera, Roche) that was designed by chemically conjugating a large PEG moiety (>30 kDa) site-specifically to recombinant EPO, resulting in a plasma half-life of 134 h after intravenous administration in patients with anemia and chronic kidney disease on dialysis^9–11^.

An alternative approach could be to fuse EPO to a protein carrier that can provide prolonged plasma half-life and transcellular transport properties. In this regard, an attractive strategy is to genetically fuse EPO to human albumin or immunoglobulin G (IgG), both possessing an unusually long plasma half-life of about 3 weeks in humans^12^. The reason for this is their ability to engage the broadly expressed neonatal Fc receptor (FcRn)^13,14^, which rescues both ligands from intracellular degradation via a recycling process^13,15–19^. Mechanistically, albumin and IgG enter the endothelial cells that line blood vessels through fluid-phase pinocytosis. Here, they encounter FcRn, which is predominantly situated in acidified endosomes. The pH of the endosomes (pH 5–6) facilitates binding of the ligands^20–22^, which then follow the receptor to the cell surface, where exposure to the near-neutral pH of the blood results in release of the ligands into the extracellular space^21–24^. Thus, FcRn-mediated recycling ensures that albumin and IgG avoid lysosomal degradation. An in-depth understanding of this biology inspires the design of carrier fusion proteins with favorable pharmacokinetic properties.

FcRn also mediates bidirectional and pH-dependent transcytosis of the ligands across polarized epithelial cell layers at mucosal barriers, such as that of the lungs, after intranasal delivery^18,19,25^. Furthermore, we have demonstrated that albumin is transcytosed more efficiently than IgG from the outer luminal side to the inner basolateral side when given intranasally to human FcRn transgenic mice^19^. As non-invasive delivery of complex proteins across mucosal barriers normally is hampered by poor uptake and delivery to tissue and blood, it follows that FcRn is an attractive receptor to target for the delivery of albumin-fused therapeutic proteins across mucosal surfaces.

EPO has been genetically fused to the N-terminal end of human IgG1 fragment crystallizable (Fc) (EPO-Fc) in a monovalent manner, resulting in a plasma half-life of 25 h compared to 6.5 h for epoetin alfa (Epogen, Amgen) in cynomolgus monkeys^26^. When an aerosolized version was delivered directly into the lungs of the animals under breathing controlled by a respirator, above 2-fold more EPO was detected in sera of the cynomolgus monkeys given the Fc fusion compared to epoetin alfa^26^. Moreover, healthy adults who received an EPO-Fc homodimer via pulmonary administration showed a dose-dependent amount of fusion in the circulation and an increased number of erythrocytes for the highest dosed group^27^. EPO fused to the C-terminal end of wild-type (WT) human albumin has been tested in rats and the plasma half-life profile was found to be the same as that of darbepoetin alfa^28^. This is not surprising, as human albumin binds poorly to mouse and rat FcRn^29–31^ and human albumin fusions administered to rodents will be outcompeted for FcRn binding by large amounts of endogenous albumin. Thus, the potential of human albumin as a carrier for EPO has not been fully explored.

Here, we report on an EPO fused to a human albumin variant engineered for improved pH-dependent binding to human FcRn^19^. The fusion showed equal ability to engage the EPO receptor (EPOR) and to mediate cell proliferation as that of a commercial EPO product, both in vitro and in mice. Importantly, favorable FcRn binding kinetics translated into extended plasma half-life in human FcRn-expressing mice. In addition, we demonstrate enhanced transport across polarized mucosal cell barriers for the albumin-engineered EPO fusion, resulting in proliferation of erythrocytes after intranasal delivery in mice.

## Results

### EPO-fused human albumin variants yield pure monomeric fusions

**Fig. 1:**
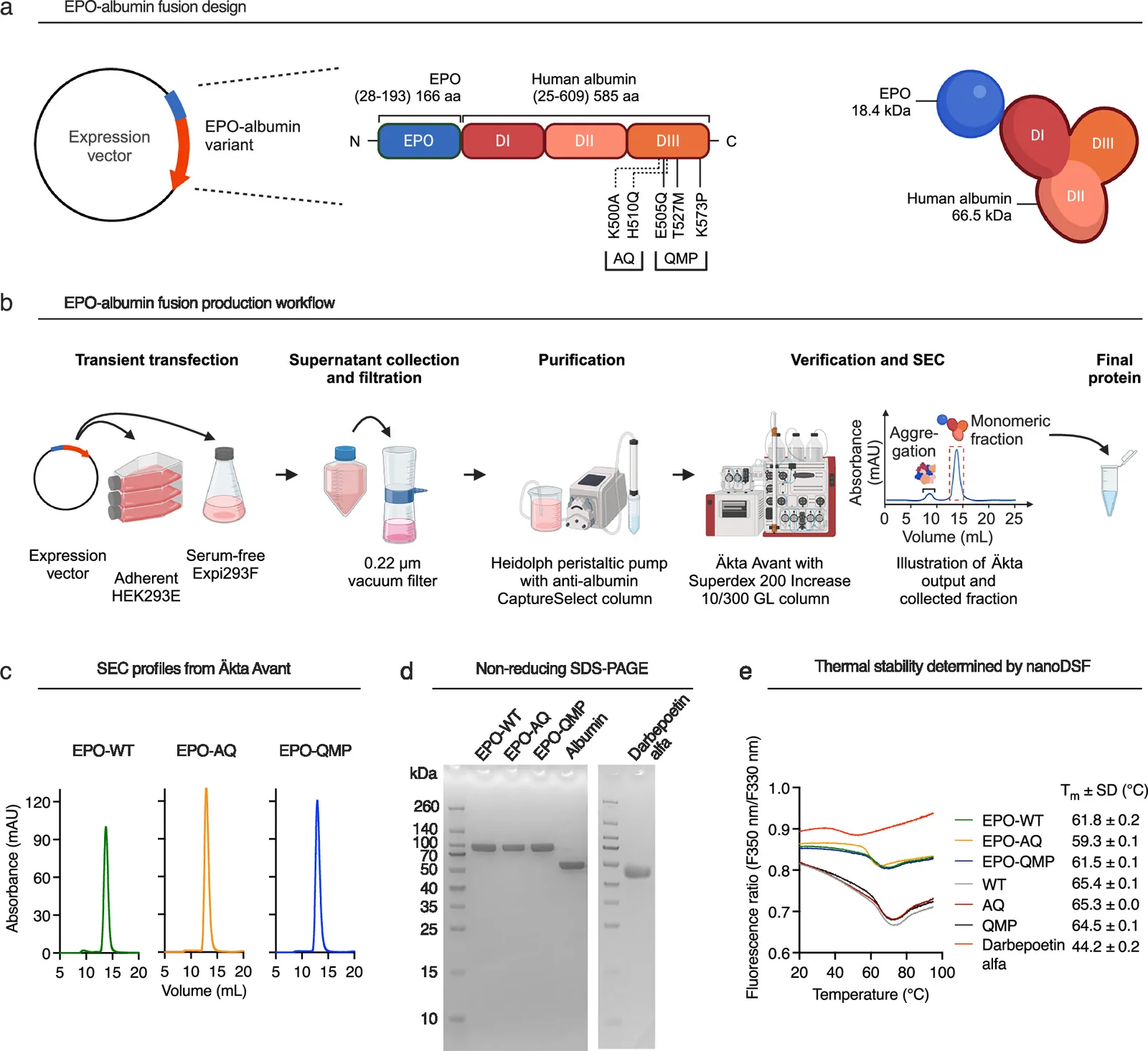
EPO-fused human albumin variants produced as stable monomeric fusions. **a** A schematic representation of the expression vector with cDNA encoding the recombinant human EPO-albumin fusion proteins containing WT, AQ, and QMP albumin. The number of amino acids (aa) of human EPO and human albumin is indicated. **b** Illustration of the production workflow that includes transient transfection of adherent HEK293E cells or Expi293F cells in serum-free suspension, followed by purification on anti-human serum albumin CaptureSelect columns connected to a peristaltic pump and isolation of pure monomeric fractions by SEC. **c** Representative SEC profiles of purified EPO-albumin fusions. **d** Migration of the purified monomeric EPO-albumin fusions, unfused WT albumin, and darbepoetin alfa on a representative non-reducing SDS-PAGE gel from three independent gels. The theoretical molecular masses are 84.9 kDa for the EPO-albumin fusions, 66.5 kDa for unfused albumin, and 37.0 kDa for darbepoetin alfa. **e** Thermal stability of the EPO-albumin fusions, unfused albumin variants, and darbepoetin alfa was measured using nanoDSF. Curves represent each replicate of triplicates, and *T*_*m*_ values are indicated as mean ± SD. **a**, **b** created in BioRender. Anthi, A. (2026) https://BioRender.com/7faobuw. Source data are provided as a Source Data file.

To design recombinant EPO-albumin fusions, human EPO was fused directly to the N-terminal end of human albumin variants (Fig. 1a). Briefly, cDNA corresponding to human EPO was genetically fused in frame with complementary DNA (cDNA) encoding a full-length WT albumin (EPO-WT), an engineered human albumin variant containing three amino acid substitutions (E505Q/T527M/K573P; QMP) (EPO-QMP), which has been shown to improve pH-dependent binding to human FcRn 180-fold^19^, and a variant engineered for reduced binding to the receptor (K500A/H510Q; AQ) (EPO-AQ) (Fig. 1a). All three expression vectors as well as previously made vectors encoding unfused WT human albumin, QMP and AQ were transiently transfected into adherent HEK293E cells followed by collection and replacement of medium every 2–3 days for up to 2 weeks. The collected supernatants were filtered and run through anti-human serum albumin CaptureSelect columns connected to a peristaltic pump system, and the captured proteins were eluted as described in “Methods”, followed by size-exclusion chromatography (SEC) to isolate monomeric fractions (Fig. 1b, c). When applied on a non-reducing SDS-PAGE gel, the purified EPO-albumin fusions (84.9 kDa) and the commercial unfused EPO product darbepoetin alfa (Aranesp; 37.0 kDa) migrated with expected molecular mass, as did the unfused WT albumin (66.5 kDa) (Fig. 1d). To ease production, we adapted the protocol to a serum-free system where Expi293F cells were grown in suspension and produced protein with the same characteristics (Supplementary Fig. 1).

Next, the thermal stability of the recombinant proteins was determined by nano differential scanning fluorimetry (nanoDSF), which revealed similar melting temperatures (*T*_*m*_) for the EPO-albumin fusions (59.3–61.8 °C), slightly lower than that of the corresponding unfused albumin variants (65.4–64.5 °C), and about 16 °C higher than that of darbepoetin alfa (44.2 °C) (Fig. 1e). Thus, fusion of EPO to the human albumin variants did not compromise secretion from the cells or thermal stability.

### Albumin-fused EPO induces erythrocyte proliferation

**Fig. 2:**
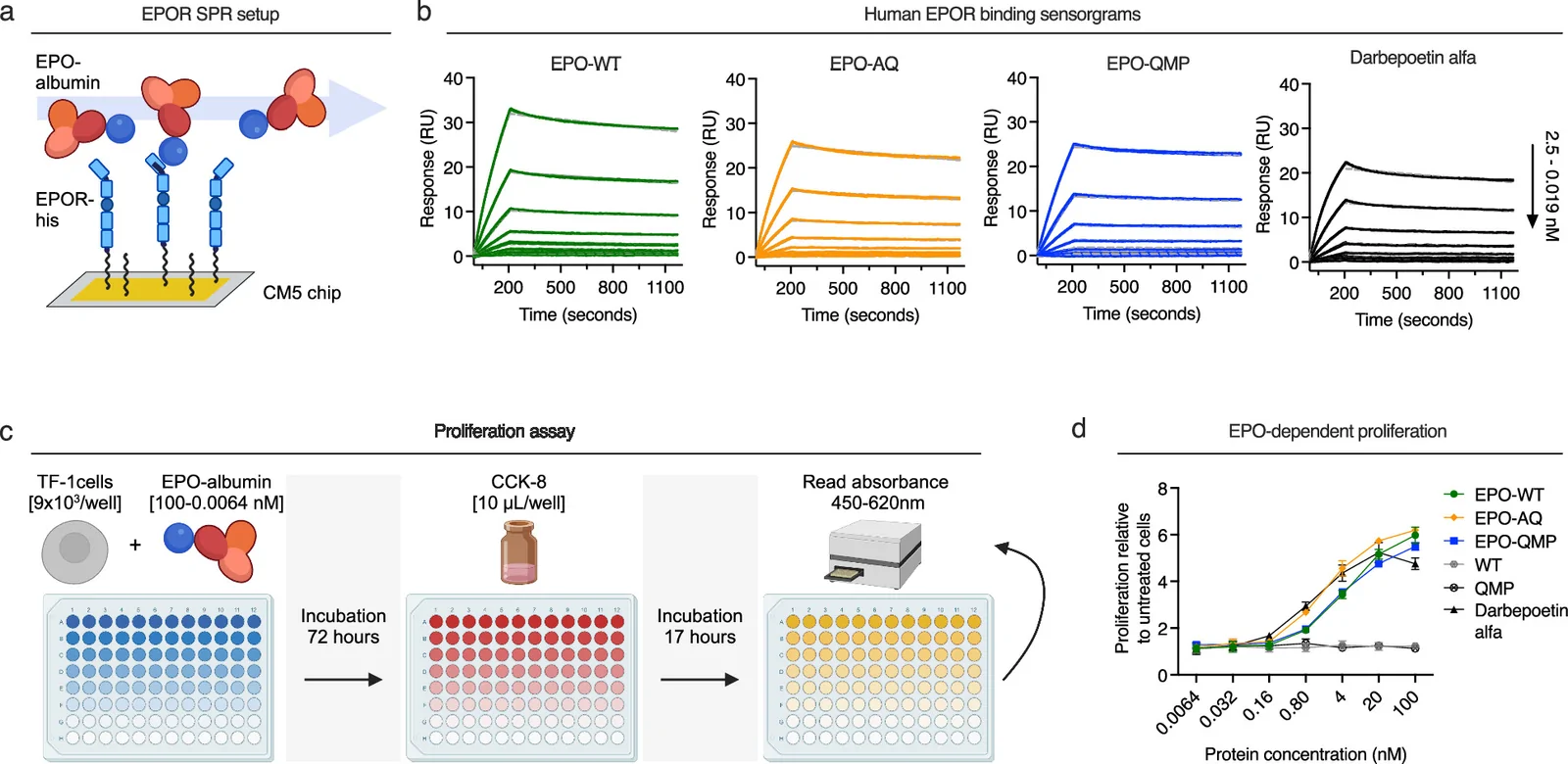
EPOR binding and cell proliferation properties of the EPO-albumin fusions. **a** Illustration of the SPR setup for measuring the binding kinetics between human EPOR and EPO fused to the N-terminal end of human albumin variants. EPOR was covalently immobilized by amine coupling chemistry to a CM5 sensor chip (~ 200 RU), followed by injection of the monomeric EPO-albumin fusions or darbepoetin alfa. **b** The resulting SPR sensorgrams of 2-fold serial dilutions (2.5–0.019 nM) of the EPO-albumin variants (WT, AQ, and QMP) or darbepoetin alfa injected in duplicates over immobilized human EPOR, where each replicate is presented as a solid line. Curves fitted to the 1:1 Langmuir binding model are presented as dashed lines. **c** Illustration of the cellular EPO-dependent proliferation assay using the human TF-1 cell line that was exposed to serial titrations (100–0.0064 nM) of the EPO-human albumin fusion variants, unfused albumin variants (WT and QMP), or darbepoetin alfa, followed by adding CCK-8. **d** The resulting cell proliferation values for the cells exposed to the different proteins relative to untreated cells, presented as mean ± SD of *n* = 6 for EPO-WT and EPO-QMP, *n* = 4 for EPO-AQ, *n* = 2 for WT and QMP, and *n* = 8 for darbepoetin alfa, for each protein concentration, from one independent experiment repeated once. **a**, **c** created in BioRender. Anthi, A. (2026) https://BioRender.com/7faobuw. Source data are provided as a Source Data file.

To investigate if the EPO-fused albumin fusions could interact with the EPOR, we used surface plasmon resonance (SPR) with a recombinant form of the human receptor immobilized by amine coupling to a CM5 chip (Fig. 2a). Injection of serial titrations of SEC-purified monomeric EPO-albumin fusions resulted in concentration-dependent binding (Fig. 2b). The sensorgrams were fitted to a Langmuir 1:1 binding model, and the derived binding kinetics revealed sub-nM equilibrium dissociation constant (KD) for all three EPO-fused albumin variants (0.09–0.12 nM), in range with that measured for darbepoetin alfa (0.07 nM) (Table 1). In addition, we measured binding to the mouse form of EPOR, which displays 82% similarity in amino acid sequence to the human version (Supplementary Fig. 2). The result, again, was sub-nM KD values for the interactions (KD: 0.37–0.91 nM) (Supplementary Table 1 and Supplementary Fig. 3a, b).

**Table 1:** SPR-derived binding kinetics for EPO-albumin fusions for human EPOR and human FcRn

	Human EPOR				Human FcRn				
Variant	*k*_*a*_ ± SE (×10^4 ^M⁻¹ s⁻¹)	*k*_*d*_ ± SE (×10⁻^5^ s⁻¹)	KD (×10⁻⁹ M)	Chi² (RU²)	*k*_*a*_ ± SE (×10^4 ^M⁻¹ s⁻¹)	*k*_*d*_ ± SE (×10⁻^5^ s⁻¹)		KD (×10⁻⁹ M)	Chi² (RU²)
EPO-WT	164.000 ± 0.146	14.700 ± 0.015	0.090	0.041	3.650 ± 0.009	833.000 ± 1.580		229.000	0.822
EPO-AQ	152.000 ± 0.061	14.000 ± 0.014	0.092	0.024	ND				
EPO-QMP	70.100 ± 0.149	8.390 ± 0.021	0.120	0.059	12.000 ± 0.004	8.740 ± 0.004		0.727	3.920
QMP	NA				10.700 ± 0.007	10.800 ± 0.092		1.010	2.150
Darbepoetin alfa	229.000 ± 0.308	16.800 ± 0.019	0.073	0.025	NA				
EPO-QMP -Expi293F	660.000 ± 2.600	23.100 ± 0.082	0.035	0.099	12.000 ± 0.004		12.400 ± 0.005	1.040	3.810

Kinetic parameters were obtained by global fitting of all concentrations, including duplicate injections, using a 1:1 Langmuir binding model. Standard errors (SE) are derived from the covariance matrix of the fit.

*ND* not detected, *NA* not analyzed.

Next, we investigated whether the EPO-albumin fusions could induce cell proliferation by engaging EPOR. To do so, the human erythroblast cell line TF-1 with endogenously expressed EPOR was grown in suspension before exposure to serial titrations of equimolar amounts of the EPO-albumin fusions or darbepoetin alfa (Fig. 2c). In short, cells were spun and seeded in fresh medium containing titrated amounts of the EPO-fused albumin variants, unfused albumin, or darbepoetin alfa, followed by incubation for 3 days. Cells grown without the addition of any of the proteins served as a reference. The number of live cells was determined by colorimetric absorbance at OD 450–620 nm after an overnight incubation with 10 μL/well of cell counting kit-8 (CCK-8), where the amount of formazan produced is directly proportional to the number of living cells. The rate of proliferation was calculated by dividing the OD values for the stimulated cells by the average OD values for the unstimulated cells for each concentration of EPO-albumin fusion. The results showed that the three EPO-albumin fusions induced proliferation of the TF-1 cells to a similar extent as darbepoetin alfa, while albumin alone (WT and QMP) did not affect proliferation (Fig. 2d). Hence, fusion of EPO to the N-terminal end of full-length human albumin allows efficient engagement of its receptor and results in proliferation of a human erythroblast cell line.

### EPO fusion to albumin does not compromise pH-dependent FcRn binding

**Fig. 3:**
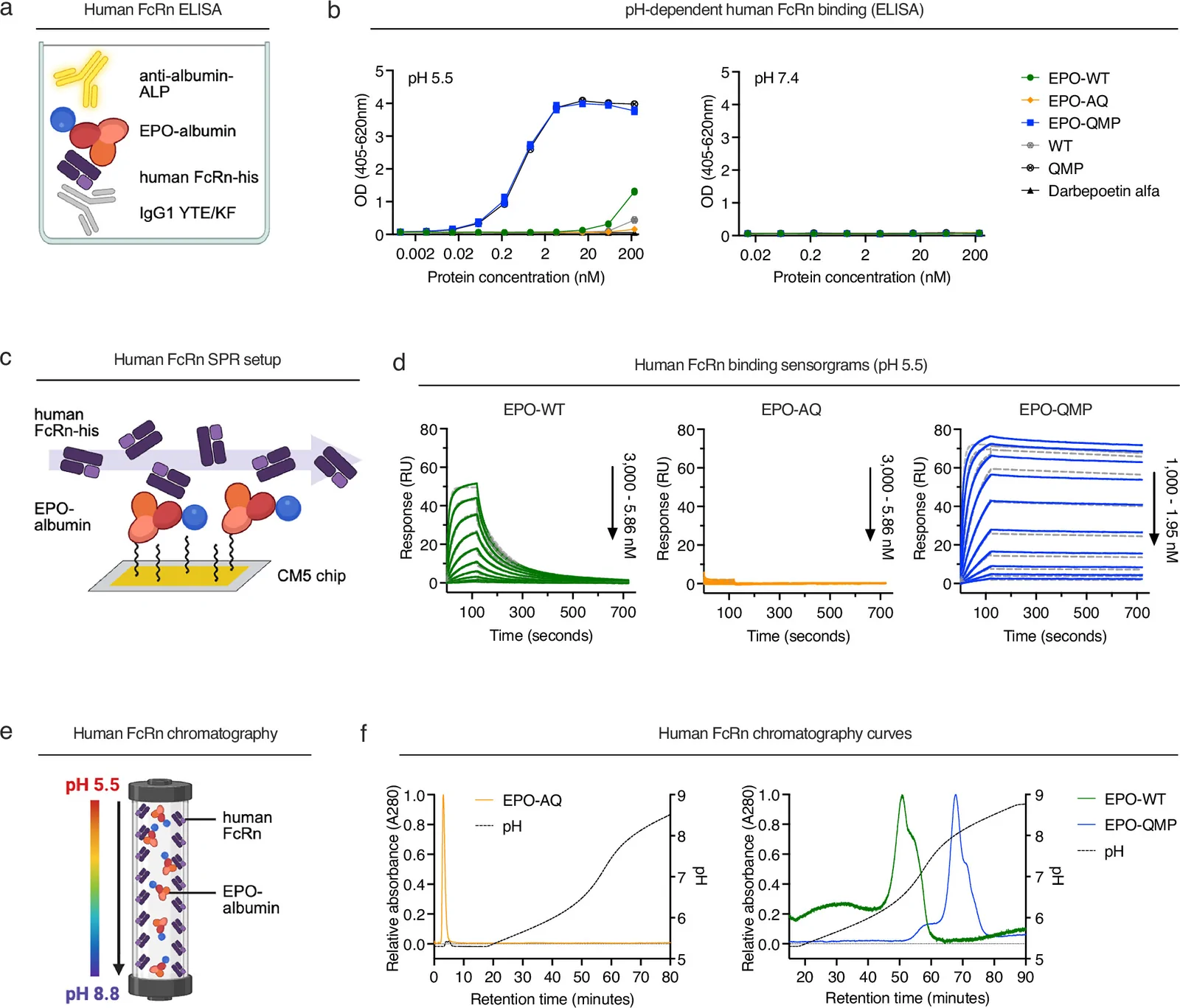
Human FcRn binding properties of EPO-albumin fusion variants. **a** Illustration of the ELISA used to study FcRn binding, where the albumin variants were captured on human FcRn bound to a human IgG1 variant (YTE/KF) coated in the wells. Bound albumin was detected with alkaline phosphatase (ALP)-conjugated anti-human albumin antibody. **b** The resulting binding curves at pH 5.5 (each replicate of duplicates) and pH 7.4 (singlets) for the EPO-fused albumin variants (WT, AQ, and QMP) and unfused WT and QMP albumin run in 4-fold serial dilution (236.0–0.0009 nM). **c** Illustration of the SPR setup used to derive binding kinetics between the albumin variants and human FcRn, where the EPO-albumin fusion variants were immobilized via amine coupling chemistry to a CM5 sensor chip (~ 200 RU). **d** The resulting SPR sensorgrams obtained at pH 5.5 following injection of 2-fold serial titrations of human FcRn run in duplicates (at 3000–5.86 nM for EPO-WT and EPO-AQ, and 1000–1.95 nM for EPO-QMP), where each replicate is presented as a solid line. The binding curves were fitted to the 1:1 Langmuir binding model and presented as dashed lines. **e** Illustration of the analytical human FcRn chromatography used to measure pH-dependent release of human albumin variants (154 μg injected) from the receptor-coupled matrix when exposed to buffer with increasing pH (5.5–8.8). **f** The resulting binding and release profiles of the EPO-albumin variants as relative absorbance and as a function of the pH gradient. **a**, **c**, **e** created in BioRender. Anthi, A. (2026) https://BioRender.com/7faobuw. Source data are provided as a Source Data file.

To assess binding of the EPO-fused albumin variants to human FcRn, an enzyme-linked immunosorbent assay (ELISA) was established, where a recombinant truncated soluble form of the receptor, containing its extracellular domains, was captured on a human IgG1 variant (YTE/KF) engineered for strong binding at slightly acidic as well as neutral pH, which was coated in the wells (Fig. 3a). Equimolar amounts of the EPO-albumin fusions, the unfused albumin variants, and darbepoetin alfa were added, followed by detection of bound albumin using an anti-human albumin antibody. The results revealed strong and concentration-dependent binding of the EPO-QMP variant at pH 5.5, equal to that of unfused QMP, and weak binding of the WT albumin variants (Fig. 3b). The EPO-AQ fusion protein showed weak binding, only detectable at the highest concentration (Fig. 3b). At pH 7.4, none of the formats showed detectable binding to the receptor at the tested concentrations (Fig. 3b).

To gain insight into the binding kinetics, we took advantage of an SPR setup where EPO-albumin fusions and controls were immobilized by amine coupling on CM5 chips before titrated amounts of monomeric truncated human FcRn were injected at pH 5.5 (Fig. 3c). Fitting the sensorgrams to the 1:1 Langmuir binding model resulted in a KD value of 0.7 nM for the EPO-QMP, similar to that of unfused QMP (1.0 nM), and much stronger than EPO-WT (228.5 nM) (Table 1, Fig. 3d and Supplementary Fig. 4a). In line with the ELISA results, only negligible binding responses were observed for the EPO-AQ variant (Table 1, Fig. 3d and Supplementary Fig. 4a).

Furthermore, we measured FcRn binding and release of the EPO-albumin fusions as a function of pH by analytical human FcRn affinity chromatography. The system mimics receptor release during the cellular exocytic process. The assay was performed by injecting the proteins on to the column in a buffer of pH 5.5 and study their retention to human FcRn coupled to the column as the pH gradually increased from 5.5 to 8.8 (Fig. 3e). The results demonstrated that EPO-WT was released at a similar pH as unfused WT albumin, at pH 6.5 and pH 6.3, respectively. EPO-QMP was released at the same pH as unfused QMP albumin, at pH 8.0, and importantly, the QMP-containing variants were released at a higher pH than the WT counterparts (Table 2, Fig. 3f and Supplementary Fig. 4b). Thus, fusion of EPO did not alter the pH-dependent FcRn binding of the albumin variants. As expected, the AQ-containing variants were ignored by the receptor-coupled matrix and, as such, eluted immediately after being applied to the column (Table 2, Fig. 3f and Supplementary Fig. 4b).

**Table 2:** Analytical human FcRn chromatography on EPO-albumin fusions and unfused albumin counterparts

Variant	Elution pH
EPO-WT	6.5
EPO-AQ	ND
EPO-QMP	8.0
WT	6.3
AQ	ND
QMP	8.0
EPO-QMP -Expi293F	8.0

### Albumin engineering leads to enhanced FcRn recycling and plasma half-life

**Fig. 4:**
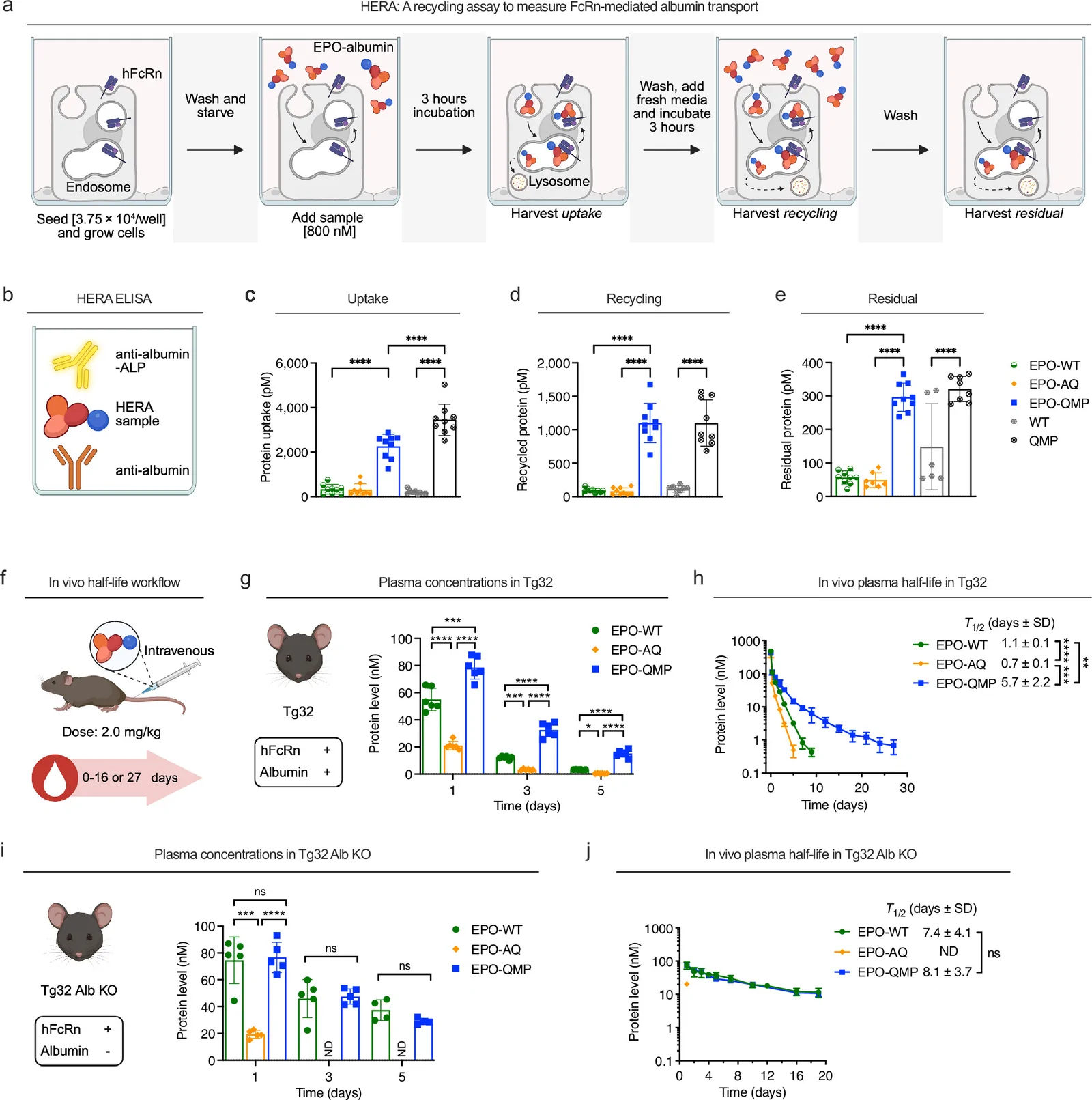
Human FcRn rescues EPO-albumin from intracellular degradation, and competition for receptor binding affects plasma half-life. **a** Illustration of the HERA protocol used to measure how FcRn-expressing cells handle human albumin variants. The cells were seeded and exposed to EPO-albumin fusions or unfused albumin variants (WT and QMP) (800 nM), before cells were harvested for uptake, media harvested for recycling, and cells harvested for residual amounts at termination. **b** The two-way anti-human albumin ELISA setup used to quantify the amounts of albumin in the harvested HERA samples. Results from **c** uptake, **d** recycling and **e** residual samples (mean ± SD) of three independent experiments run with technical triplicates. **f** The experimental setup for the pharmacokinetic study of EPO-albumin fusions delivered intravenously at 2 mg/kg in human FcRn transgenic Tg32 mice, in the **g**, **h** presence (Tg32, *n* = 6 (3 M/3 F)) or **i**, **j** absence (Tg32 Alb KO, *n* = 5) of endogenous mouse albumin. The protein amounts in plasma shown as mean ± SD **g**, **i** at selected timepoints visualized as dotted bar graphs and **h**, **j** at all time points visualized as clearance curves. Plasma concentrations were quantified using a two-way anti-albumin ELISA and the graphs show all values interpolated using Prism, where values below lower limit of detection (LLOD) and those Prism cannot interpolate were defined as not detected (ND). The mean plasma half-life (*T*_1/2_ ± SD) are shown in days. Statistical details and exact *p* values are listed in Supplementary Table 2. **c**–**g**, **i** (day 1) two-tailed one-way ANOVA with Tukey’s multiple comparison, and **h**, **i** (day 3 and 5) two-tailed unpaired *t*-test. ****p ≤ 0.0001; ***p  ≤  0.0010; **p ≤ 0.0100; *p ≤ 0.0500; ns *p* > 0.0500. hFcRn human FcRn. **a**, **b**, **f** created, or **g**, **i** partially created in BioRender. Anthi, A. (2026) https://BioRender.com/7faobuw. Source data are provided as a Source Data file.

To address recycling of the EPO-fused albumin variants, we used a human endothelial cell-based recycling assay (HERA)^32^. Briefly, the adherent HMEC-1 cell line, which over-expresses human FcRn, was exposed to the EPO-albumin fusions as well as unfused WT or QMP albumin for 3 h (Fig. 4a). Subsequently, the medium was removed, and cells were washed and lysed to measure the amounts present inside the cells (defined as uptake sample). In parallel, cells on a separate plate were washed and incubated for an additional 3 h in fresh recycling medium. The medium was then collected to measure the amounts that were released from the cells (defined as recycling sample), while the remaining cells were lysed to measure the amounts remaining inside the cells at termination of the experiment (defined as residual sample). Protein quantification by a two-way anti-albumin ELISA (Fig. 4b) revealed 5-fold more of the EPO-QMP than the EPO-WT inside the cells after the initial incubation step, while the difference was 13-fold between the unfused albumin variants (Fig. 4c). The amount released from the cells was almost the same for the QMP-containing variants, 10–11-fold higher than that of the WT albumin variants (Fig. 4d). At termination of the experiment, the amounts remaining inside the cells were about 5-fold higher for the QMP-containing variants compared to EPO-WT and WT (Fig. 4e). The amount of EPO-AQ was slightly lower than that of EPO-WT (Fig. 4c–e). No statistical differences were observed between the EPO-albumin fusions and the unfused albumin variants, except for the higher uptake of unfused QMP compared to EPO-QMP (Fig. 4c and Supplementary Table 2).

Next, to study how FcRn binding translated into in vivo pharmacokinetic properties in the presence of mouse EPOR, we delivered the three EPO-albumin variants (WT, AQ, and QMP) to human FcRn transgenic mice through intravenous administration (2.0 mg/kg), followed by collection of blood samples and isolation of plasma for up to 27 days (Fig. 4f). The levels of EPO-albumin fusions in the plasma were quantified by a two-way albumin ELISA. The results from the study in mice expressing human FcRn (Tg32) revealed that similar amounts were detected of EPO-WT and EPO-QMP at both 5 min and 8 h post injection, with a 4-fold drop in amounts between the timepoints (Supplementary Fig. 4d). At 24 h, the amounts of EPO-WT and EPO-QMP dropped further by 8.7- and 5.4-fold, respectively, compared to that measured at 5 min. Over time, higher amounts of EPO-QMP were detected than the WT counterpart, with 1.4-fold more at 24 h, 2.7-fold on day 3, and 4.8-fold on day 5 post injection (Fig. 4g, h). On day 5, EPO-QMP was detected at 28-fold lower amounts than after 5 min, while the difference for EPO-WT was as much as 150-fold (Fig. 4g and Supplementary Fig. 4d). The EPO-AQ variant, engineered for reduced FcRn binding, was detected in similar amounts as the other EPO fusions at 5 min but decreased by 7.1-fold at 8 h and as much as 700-fold over the following 5 days, after which the variant was not detected (Fig. 4g, h and Supplementary Fig. 4d). Notably, EPO-QMP could be detected for up to 27 days post administration while EPO-WT levels were not detectable after 10 days. As such, the results demonstrated a profound effect of albumin engineering for improved and reduced FcRn engagement.

To derive the plasma half-life values and pharmacokinetic parameters, a non-compartmental analysis (NCA) was performed on the measured plasma concentrations using the gPKPDSim for MATLAB^33^. The results showed the slowest clearance for EPO-QMP (60.3 ± 9.3 mL/d/kg), followed by a 1.6-fold faster clearance for the WT counterpart (97.4 ± 6.1 mL/d/kg) (Table 3), while EPO-AQ was cleared rapidly (193.9 ± 30.0 mL/d/kg) (Table 3). The plasma half-life of the QMP-containing variant (5.7 ± 2.2 days) was 5.2-fold longer than that of EPO-WT (1.1 ± 0.1 days) (Fig. 4h, Supplementary Fig. 4c and Table 3).

**Table 3:** Non-compartmental pharmacokinetic analysis after intravenous administration in human FcRn transgenic mice

Mouse strain	Fusion variant	Route	AUC	*C*_max_	CL	MRT	*V*_ss_	*T*_1/2_	*R*^2^	Bioavailability
			(nM × d)	(nM)	(mL/kg/d)	(d)	(mL/kg)	(d)		
Tg32	EPO-WT	i.v.	242.3 ± 15.5	476.5 ± 60.0	97.4 ± 6.1	1.0 ± 0.1	94.4 ± 12.3	1.1 ± 0.1	0.985	
	EPO-AQ	i.v.	123.5 ± 17.9	368.2 ± 64.5	193.9 ± 30.0	0.5 ± 0.0	99.7 ± 15.8	0.7 ± 0.1	0.998	
	EPO-QMP	i.v.	392.6 ± 56.4	424.3 ± 48.7	60.3 ± 9.3	3.6 ± 0.7	212.9 ± 28.3	5.7 ± 2.2	0.966	
		s.c.	237.8 ± 42.6	63.3 ± 11.9	100.7 ± 18.8	4.3 ± 1.1	–	4.9 ± 1.3	0.964	76%
Tg32 Alb KO	EPO-WT	i.v.	488.8 ± 212.3	74.5 ± 18.6	33.1 ± 4.2	9.3 ± 4.2	355.1 ± 55.1	7.4 ± 4.1	0.9243	
	EPO-AQ	i.v.	ND	ND	ND	ND	ND	ND	ND	
	EPO-QMP	i.v.	473.4 ± 147.3	76.6 ± 11.3	40.0 ± 12.0	10.0 ± 4.2	632.5 ± 91.7	8.1 ± 3.7	0.981	

Pharmacokinetic parameters for EPO-WT, EPO-AQ, and EPO-QMP administered to Tg32 or Tg32 Alb KO mice (2 mg/kg; 23.6 nmol/kg) by either intravenous (i.v.) or subcutaneous (s.c.) injection were calculated by applying the plasma concentration-time data in Fig. 4h, j, and in Supplementary Fig. 4f to the gPKPDSim for MATLAB. Data are presented as the mean ± SD (Tg32, *n* = 6; Tg32 Alb KO, *n* = 5).

*i.v.* intravenous, *s.c.* subcutaneous, *AUC* area under the curve from time 0 to the last measured time point, *C*_*max*_ maximum concentration following administration, *CL* clearance, *MRT* mean residency time, *V*_*ss*_ volume of distribution at steady state, *T*_*1/2*_ plasma half-life, *R*^*2*^ coefficient of determination, *Bioavailability* calculated using formula: (AUC_SC,8h-27days_ x Dose_IV_) / (AUC_IV,8h-27days_ x Dose_SC_), *NA* not analyzed due to insufficient number of data points.

Furthermore, we addressed how a subcutaneous delivery route would affect the plasma half-life and pharmacokinetics of EPO-QMP with the same dose as before (2.0 mg/kg). While the amounts detected after intravenous administration decreased 1.4-fold between 8 h and 24 h, specifically from 111.8 to 78.4 nM, subcutaneous administration resulted in a 1.7-fold increase, from 37.7 to 63.3 nM in the same interval (Supplementary Fig. 4e). For the timepoints following 24 h, comparable reductions in detected EPO-QMP were observed for both administration routes (Supplementary Fig. 4e, f). Mice given EPO-QMP by the subcutaneous route showed a maximum plasma concentration (C_max_) of 63.3 nM, 6.7-fold lower than that measured after intravenous administration (Table 3). In addition, the time point at which C_max_ was measured was shifted, from 5 min with intravenous administration to 24 h with subcutaneous administration (Supplementary Fig. 4d–f). Despite the differences in amounts detected at early time points, the plasma half-life after subcutaneous administration (4.9 ± 1.3 days) was not statistically different from that after intravenous administration (Table 3, Supplementary Fig. 4f and Supplementary Table 2).

The potential generation of antibodies against the human EPO-albumin fusions or unfused QMP albumin in Tg32 mice was evaluated by ELISA. Specifically, we compared plasma samples collected at early time points (5 min for intravenous and 8 h for subcutaneous) with those of samples collected on day 27. The results showed no sign of immunogenicity raised towards either the EPO albumin fusions (Supplementary Fig. 4g) or unfused QMP albumin (Supplementary Fig. 4h).

The study in Tg32 mice was done in the presence of large amounts of constitutively produced endogenous mouse albumin, which modulates the plasma half-life of administered human albumin fusions^19,34^. To address the effect of in vivo competition for FcRn binding, we measured the pharmacokinetic properties of the EPO-containing variants in a Tg32 mouse strain engineered to lack expression of mouse albumin (Tg32 Alb KO), again, following intravenous administration at 2.0 mg/kg (Fig. 4f, i). One day after delivery, the plasma amounts of the respective EPO-albumin fusions were measured to be similar between the mice strains (Fig. 4g, i). In contrast to the Tg32 mice, the difference between the plasma levels of EPO-QMP and EPO-WT were stable and minor over time, reflecting the lack of competition for receptor binding (Fig. 4i, j and Supplementary Fig. 4c). This resulted in similar plasma half-lives for the EPO-QMP (8.1 ± 3.7 days) and the WT counterpart (7.4 ± 4.1 days) (Table 3, Fig. 4j and Supplementary Table 2). In line with this, the clearance of EPO-QMP (40.0 ± 12.0 mL/d/kg) and EPO-WT (33.1 ± 4.2 mL/d/kg) was reduced by 2.9- and 1.5-fold, respectively, compared with the values measured in the Tg32 mice (Table 3). The AQ-containing variant was still rapidly cleared, and could not be detected from day 3, which excluded derivation of pharmacokinetic parameters for this FcRn-reduced binder (Fig. 4h–j and Table 3).

### Albumin engineering enhances transmucosal transport upon intranasal delivery

**Fig. 5:**
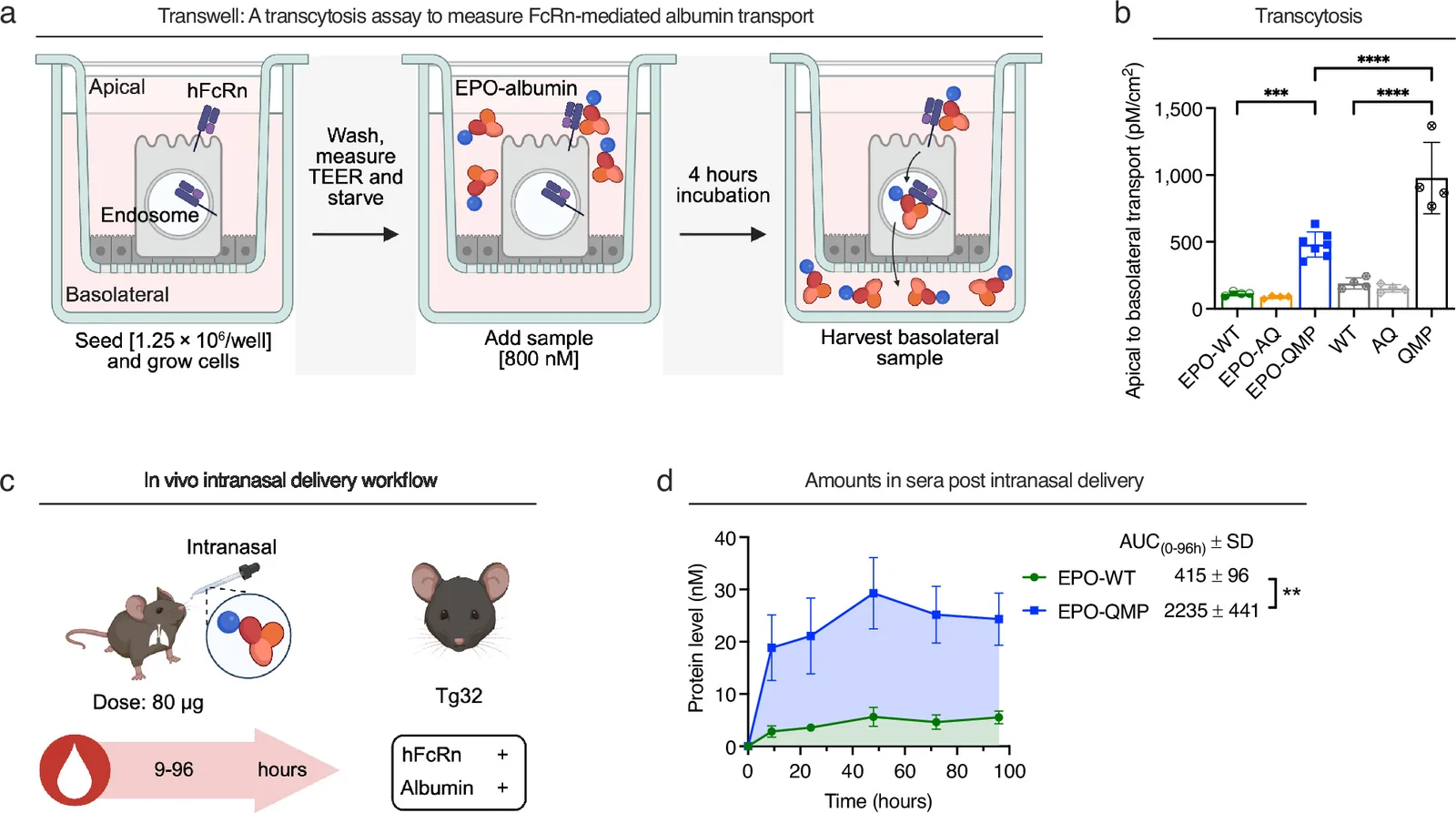
EPO-QMP shows enhanced transcellular transport both across polarized epithelial barriers and upon intranasal delivery to human FcRn-expressing mice. **a** Illustration of the transwell assay used to measure human FcRn-mediated transcytosis from the apical to the basolateral side of MDCKII cells over-expressing the human FcRn receptor. The apical side of the polarized cell layers was exposed to the EPO-albumin fusions or unfused albumin variants (WT, AQ, and QMP) (800 nM) for 4 h followed by collection of samples from the basolateral chamber. **b** A two-way anti-human albumin ELISA was used to quantify the amounts of proteins transported to the basolateral side. Data are shown as mean ± SD of *n* = 4 from one representative experiment (data from productions in both HEK293E and Expi293F are shown for EPO-QMP, thus *n* = 7). **c** The experimental setup for delivery and collection of samples upon intranasal delivery of EPO-albumin fusions (80 µg) to male human FcRn transgenic Tg32 mice. **d** The resulting amount of the fusion proteins in circulation was detected by two-way anti-albumin ELISA. Data presented as mean ± SD of *n* = 5 (male) for each time point, accompanied by total AUC values as mean ± SD. Statistical details and exact *p* values are listed in Supplementary Table 2 (**b** two-tailed one-way ANOVA with Tukey’s multiple comparison, **d** two-tailed unpaired *t*-test). ****p ≤ 0.0001; ***p  ≤  0.0010; **p ≤ 0.0100; *p ≤ 0.0500; ns *p* > 0.0500. **a**, **c** created in BioRender. Anthi, A. (2026) https://BioRender.com/7faobuw. Source data are provided as a Source Data file.

We have previously shown that human albumin can be delivered across polarized mucosal cell barriers, and that this route of transport can be enhanced by QMP^19,35^. To explore how fusion of EPO would affect transport, polarized MDCKII cells stably over-expressing human FcRn were seeded onto membrane inserts placed in a transwell system to form a polarized monolayer of cells overnight (Fig. 5a). Equimolar amounts of the unfused albumin and EPO-albumin variants were added to the apical side and incubated for 4 h. Samples of medium were then collected from the basolateral side and the two-way human albumin ELISA was again used for quantification. We observed 4.3-fold and 5.2-fold higher amounts of transcytosed EPO-QMP and unfused QMP, respectively, compared to the WT albumin counterparts (Fig. 5b). No significant differences were measured between the unfused albumin and EPO-albumin fusion variants, except for about 2-fold lower transport of EPO-QMP compared with unfused QMP (Fig. 5b and Supplementary Table 2). In line with the HERA data, only minor differences (1.2–1.3-fold) were measured between the WT and AQ albumin-containing variants (Fig. 5b).

These encouraging in vitro results motivated studies of non-invasive intranasal delivery of the EPO-albumin fusions in human FcRn transgenic Tg32 mice. Following intranasal delivery (80 μg) of EPO-QMP and EPO-WT, blood samples were collected and sera isolated (Fig. 5c). Using ELISA for quantification and then calculation of the area under the curve (AUC), we found 5-fold more EPO-QMP than EPO-WT in the circulation 9–96 h after administration (Fig. 5d). Thus, albumin engineered for improved FcRn binding enhances transmucosal transport of EPO in human FcRn expressing mice.

### EPO-QMP induces proliferation of erythrocytes in vivo

**Fig. 6:**
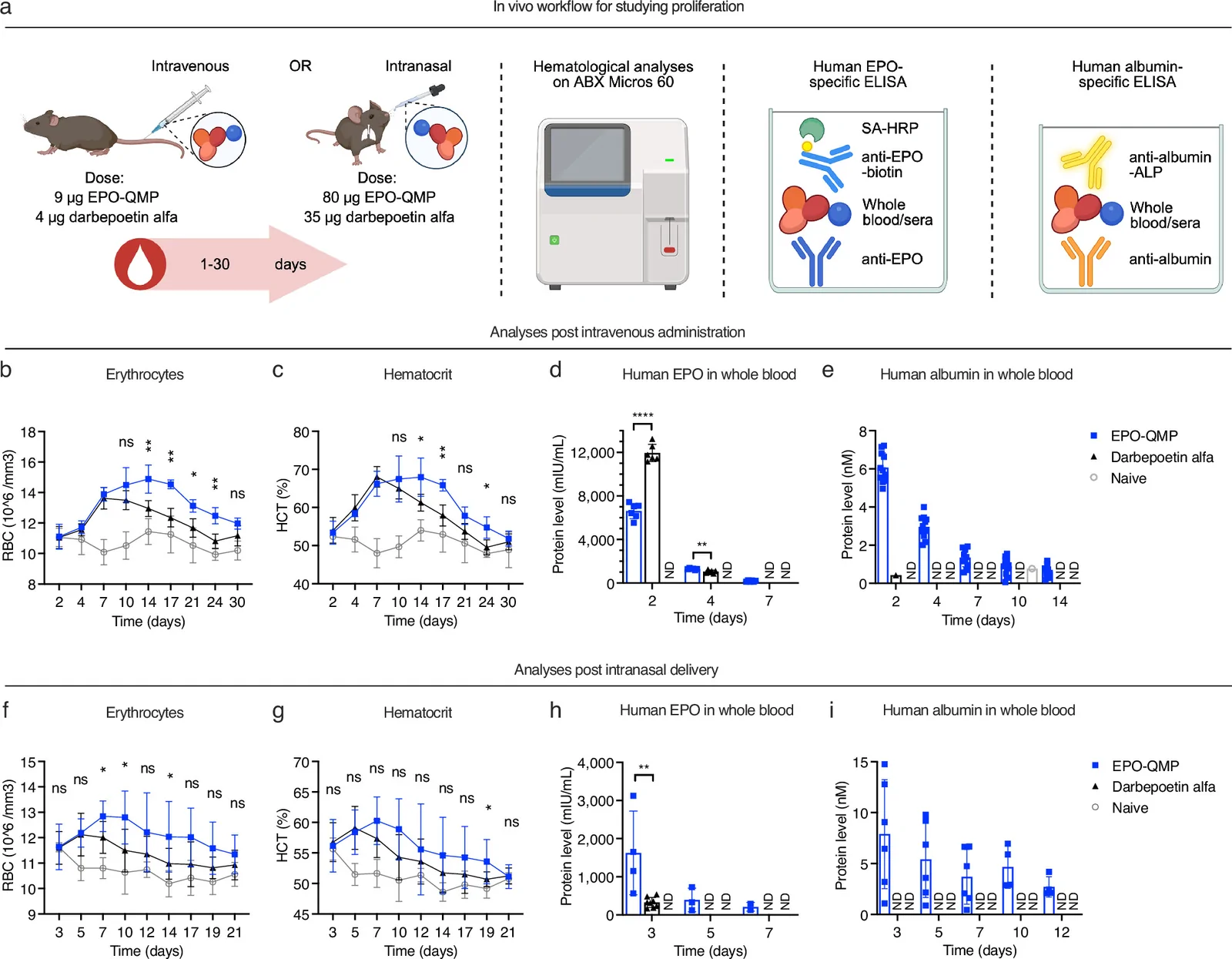
EPO-QMP induces erythrocyte proliferation upon both intravenous and intranasal delivery to mice. **a** Illustration of the workflow for intravenous and intranasal delivery of EPO-QMP or darbepoetin alfa to human FcRn transgenic mice, and the erythrocyte and hematocrit analyses done with ABX Micros 60, and the anti-human EPO and anti-human albumin ELISA setups used on collected whole blood or serum samples. Results from whole blood analyses after **b**, **c** intravenous administration (9 μg EPO-QMP or 4 μg darbepoetin alfa) or **f**, **g** intranasal delivery (80 μg EPO-QMP or 35 μg darbepoetin alfa), as **b**, **f** erythrocyte count and **c**, **g** percentage hematocrit. Protein levels in whole blood after **d**, **e** intravenous administration or **h**, **i** intranasal delivery, quantified using **d**, **h** human EPO-specific or **e**, **i** human albumin-specific ELISAs. Hematological data presented as mean ± SD for each time point, with (intravenous) *n* = 5 female per group (*n* = 3 for naive at D21-30) from one independent experiment, and (intranasal) *n* = 5 (3M/2F) for naive, *n* = 7 (3M/4F) for EPO-QMP, and *n* = 13 (5M/8F) for darbepoetin alfa from two independent experiments (samples not pooled at analysis). *p* values indicated as significance between EPO-QMP and darbepoetin alfa. Specific *p* values are listed in Supplementary Table 3. ELISA data are presented as individual samples with mean ± SD for each time point (**d** *n* = 6, 6, 2, all male (one experiment), **e** *n* = 11 (6M/5F), 11 (6M/5F), 4 (2M/2F) (two experiments), **h** *n* = 4, 8, 2, all females (one experiment), **i** *n* = 7 (3M/4F), 13 (5M/8F), 5 (3M/2F) (two experiments) for EPO-QMP, darbepoetin alfa and naive, respectively (samples not pooled at analysis), and show all values interpolated (Sigmoidal, 4PL, X is concentration) using Prism, where values below LLOD and those Prism cannot interpolate are defined as not detected (ND). Specific *p* values are listed in Supplementary Table 2 (**b**, **c**, **f**, **g** two-tailed one-way ANOVA with Tukey’s multiple comparison **d**, **h** two-tailed unpaired *t*-test). ****p ≤ 0.0001; ***p ≤ 0.0010; **p ≤ 0.0100; *p ≤ 0.0500; ns *p* > 0.0500. **a** created in BioRender. Anthi, A. (2026) https://BioRender.com/7faobuw. Source data are provided as a Source Data file.

Finally, we explored the proliferation of erythrocytes in vivo, comparing the effect of EPO-QMP and the clinical product darbepoetin alfa. Equimolar amounts of both were administered intravenously in human FcRn transgenic Tg32 mice, followed by collection of blood samples. Hematology analyses of blood samples diluted 2-fold in 2 mM EDTA were then performed on an ABX Micros 60 instrument (Fig. 6a). The results showed that both EPO-QMP and darbepoetin alfa increased the number of erythrocytes and the hematocrit count 7 days post intravenous administration (Fig. 6b, c). Subsequently, a decrease was observed in the darbepoetin alfa-treated mice, whereas the levels stayed high until day 14 in mice given EPO-QMP. By day 24, mice given EPO-QMP still had a significantly higher number of erythrocytes than the darbepoetin alfa-treated and the naive mice (Fig. 6b and Supplementary Table 3). The amounts of EPO-QMP detected in whole blood 2 days post intravenous administration were about half that of darbepoetin alfa, while at day 4 the amounts of darbepoetin alfa had decreased below that of EPO-QMP (Fig. 6d). Notably, darbepoetin alfa-treated mice displayed an 11-fold decrease in EPO-levels from day 2 to 4, compared to the 5-fold decrease measured in EPO-QMP-treated mice (Fig. 6d), reflecting the long plasma half-life of the albumin fusion. Quantifying the EPO levels in sera resulted in the same trends as observed in whole blood (Fig. 6d and Supplementary Fig. 5a), and moreover, the levels of human albumin detected in whole blood and sera were similar (Fig. 6e and Supplementary Fig. 5b).

Lastly, equimolar amounts of EPO-QMP and darbepoetin alfa were delivered intranasally to Tg32 mice (Fig. 6a). Lower amounts of EPO were detected in whole blood after intranasal delivery than after intravenous administration (Fig. 6d, h), despite detection of similar levels of human albumin (Fig. 6e, i). Again, quantification of the amounts of EPO in sera resulted in the same trends as in whole blood (Fig. 6h and Supplementary Fig. 5c), and the amounts of human albumin detected in whole blood and sera were alike (Fig. 6i and Supplementary Fig. 5d). Importantly, EPO-QMP, again, performed better than darbepoetin alfa, both regarding increase in erythrocytes and hematocrit count (Fig. 6f, g). Five-fold more EPO was found in the whole blood of mice given the albumin fusion at day 3, compared to darbepoetin alfa (Fig. 6h). Thus, compared to darbepoetin alfa, EPO fused to engineered albumin has a superior effect on erythrocyte proliferation in human FcRn-expressing mice following single administration through both intravenous and intranasal routes.

## Discussion

Recombinant human EPO exhibits a short plasma half-life of 4–11 h after intravenous administration in humans^6^, which has motivated engineering strategies to extend its systemic exposure, such as to reduce plasma clearance through increased molecular mass by introduction of N-glycan sites as for darbepoetin alfa^2,8^ or by chemical conjugation to PEG as for methoxy PEG-epoetin beta^9^. Upon intravenous administration, these strategies prolong the plasma half-life by approximately 3-fold (25.3 h) for darbepoetin alfa and 14-fold (134 h) for methoxy PEG-epoetin beta compared to unmodified recombinant human EPO, allowing for reduced dosing frequency^8,9,11^.

An alternative approach to prolong the plasma half-life of recombinant EPO is to genetically fuse it to an FcRn ligand, which will allow receptor-mediated rescue from lysosomal degradation. In line with this, we report on such an engineering strategy using a human albumin variant (QMP) with improved ability to engage human FcRn in a pH-dependent manner^19^.

The EPO-albumin fusions were well produced in human cell lines and yielded pure monomeric fractions after purification. To confirm their functional properties, we demonstrated binding to recombinant EPOR, as well as the ability to induce proliferation of a human erythrocyte cell line in vitro. We showed that EPO could be genetically fused to the N-terminal end of human albumin without drastically compromising EPOR binding, as our design bound as strongly as darbepoetin alfa in the sub-nM range. In line with this, the formats induced proliferation of the erythrocyte cell line equally well, which translated into in vivo proliferation of erythrocytes in mice, as EPO-fused albumin binds the mouse version of the EPOR.

In accordance with results obtained previously with other fusion partner(s)^19,34–37^, fusing EPO N-terminally to QMP albumin did not compromise its pH-dependent binding to human FcRn, as it bound as well as unfused QMP. Furthermore, release from an analytical human FcRn coupled column as a function of pH was similar for albumin with and without fused EPO. When studied using the cellular recycling assay HERA, the ability of FcRn expressed by the cells to rescue EPO-fused QMP albumin from lysosomal degradation was considerable. Such an advantage of QMP was also observed when FcRn-mediated transcytosis was studied across a monolayer of a model cell line overexpressing the receptor.

These data motivated an investigation of in vivo pharmacokinetic properties. To do so, we used the transgenic mouse model Tg32, where the gene encoding the mouse FcRn is replaced by the human counterpart. This is important, as we have demonstrated that human albumin binds poorly to mouse FcRn and is ignored in vivo in the presence of endogenous levels of mouse albumin^29,30^. Following intravenous administration of the EPO-albumin fusions, plasma levels of EPO-QMP were 1.4-fold higher than the WT albumin fusion at 24 h, while the difference increased to 4.8-fold at day 5. This translated into a 5.2-fold longer plasma half-life when the design was based on QMP rather than WT albumin, at 5.7 days vs. 1.1 days, respectively, and consequently a 1.6-fold reduction in clearance due to enhanced FcRn binding. In line, a plasma half-life of around 1.1 day was also reported when using WT albumin as a fusion partner to coagulation factor IX, while a 2.5-fold longer plasma half-life was observed after fusion to QMP^34^. Interestingly, while the commercial coagulation factor IX-WT albumin fusion Idelvion displayed a plasma half-life of 1.0 day in the Tg32 mice^34^, its half-life in humans is 102 h^38^. Importantly, our in vivo study was conducted in the presence of the mouse EPOR, which acts as an antigen sink after administration. The excess amounts of EPO fusion proteins will then be circulating until a free target is reached, and therefore, the presence of the EPOR affects its pharmacokinetic properties. Of note, the plasma elimination curve for EPO-QMP in the Tg32 mice (Fig. 4h) displays several phases, indicating multiple modes of clearance mechanisms.

A common route of administration for protein-based therapeutics, including those that are EPO-based, is by subcutaneous injection. As such, we compared intravenous and subcutaneous administration of EPO-QMP, which resulted in similar plasma elimination curves and derived plasma half-life values, supporting both delivery strategies as relevant for future studies.

Furthermore, we performed an in vivo study using the Tg32 Alb KO mice that are not only transgenic for human FcRn, but also engineered to lack expression of liver-produced mouse albumin. This was done to showcase the importance of the presence of a competing ligand on FcRn binding. In fact, the plasma half-life of EPO-QMP was not significantly different than that of EPO-WT in the absence of ligand competition, as opposed to the more dramatic difference observed when studied in the presence of ligand competition. This illustrates that mouse albumin competes more efficiently with WT albumin than with QMP, in line with previous observations both for unfused albumin variants and a coagulation factor IX-albumin fusion^19,34^. Notably, caution must be taken when directly comparing the pharmacokinetic measurements derived from the two studies, as the initial phase prior to 24 h was not included in the Tg32 Alb KO study design. Nevertheless, the pharmacokinetic profile of EPO-QMP is clearly improved compared with EPO-WT in the presence of competition. On the other side, EPO-AQ was rapidly cleared in the human FcRn expressing strains regardless of mouse albumin being present or not, which is as expected and in line with a previous report^19^. These studies illustrate the involvement of FcRn in determining the circulatory half-life of albumin-based drugs and support the use of human albumin, in particular engineered albumin, to improve the pharmacokinetics of protein therapeutics, such as EPO.

The potential of QMP albumin warrants further studies in, for instance, non-human primates. Furthermore, it would be interesting to compare the albumin design with that of an EPO-Fc fusion, both regarding functionality and pharmacokinetics, in model systems that take cross-species differences for both FcRn ligands into consideration. While human albumin binds weakly to mouse FcRn^29,30^ and mouse IgG1 is ignored by human FcRn, human IgG1 or derived Fc binds strongly to FcRn from both species^29,39^.

We have previously shown that more albumin than IgG is transported across polarized mucosal cell surfaces and thus, more albumin than IgG reaches the blood after intranasal administration in human FcRn transgenic mice^19,35^. Here, we explored this route for the delivery of EPO-fused albumin. Following intranasal administration, we measured 5-fold more of the QMP-containing variant than EPO-WT albumin in circulation, in accordance with the improved transcellular transport measured in the in vitro transwell system. The results further encouraged investigation of erythrocyte proliferation in vivo following this non-invasive delivery route. Indeed, EPO-QMP exhibited greater erythrocyte proliferation compared with darbepoetin alfa, consistent with the results following intravenous administration. Of note, a crucial aspect for efficacy is systemic exposure over time and thus the plasma half-life. Further studies are needed to compare the efficacy of EPO-QMP with other erythropoesis stimulating agents. Nevertheless, these promising results support that our design should be explored further with a broader dosing regimen in mice and non-human primates, combined with a tailored device for delivery.

A key aspect from a translational perspective would be to determine the safety profile of the fusion design and the potential for development of anti-drug antibodies. Notably, the WT human albumin-FIX fusion Idelvion is extensively used for prophylactic treatment of patients with hemophilia B, and neutralizing antibodies against this albumin fusion were not detected in a phase 3 clinical trial^38^. Moreover, we have demonstrated that introducing the three QMP amino acid substitutions did not change the immunogenicity profile compared to that of a WT human albumin fusion when used in the design of subunit vaccines, as no antibody responses were detected against either human albumin variants after two doses of adjuvanted WT- or QMP-based vaccines in mice expressing both human FcRn and human albumin^40^. In line, antibodies against the EPO-albumin fusion or against QMP albumin were not detected in the plasma samples collected from Tg32 mice on day 27. These findings support a favorable safety profile of therapeutic fusion designs built on WT- and QMP-containing albumin.

In conclusion, EPO fused to QMP albumin should be an attractive format to explore for anemic conditions. In addition to traditional invasive delivery strategies, our results also open up for delivery across mucosal barriers. For instance, for patients in need of long-term or lifelong treatment, the benefit of a less frequent dosing regimen is indispensable. Moreover, the possibility of self-medication at home is beneficial. Thus, engineered albumin may be an attractive carrier for EPO, as well as other fusion partners where less frequent dosing and improved patient compliance are crucial.

## Methods

### Cell cultures

Semi-adherent High Five cells (Invitrogen, B85502) were cultured in flat culture flasks with Express Five SFM Medium (Gibco) supplemented with 18 mM L-glutamine (Sigma-Aldrich) and 1% Antibiotic-Antimycotic (Sigma-Aldrich) at 27 °C. Adherent human embryonic kidney (HEK) 293E cells (ATCC, CRL-1573) were cultured in RPMI 1640 (Sigma-Aldrich) supplemented with 10% heat-inactivated fetal bovine serum (FBS) and 25 U/mL penicillin streptomycin (PS) (all from Sigma-Aldrich). The cells were grown in a humid atmosphere of 5% CO_2_ at 37 °C. Expi293F cells (Gibco, A14527) were cultured in Expi293 Expression Medium (Gibco) supplemented with 25 U/mL PS. The cells were grown in shaker flasks at 125 rpm orbit shaker in a humid atmosphere of 8% CO_2_ at 37 °C. Human microvascular endothelial cells (HMEC1) stably over-expressing HA-human FcRn-EGFP (HMEC1-hFcRn) were grown in MCDB 131 medium (Gibco) supplemented with 10% heat-inactivated FBS, 2 mM L-glutamine, 25 U/mL PS, 10 ng/mL mouse epidermal growth factor (Gibco), and 1 µg/mL hydrocortisone (Sigma-Aldrich). In addition, 100 µg/mL G418 (Sigma-Aldrich) and 50 µg/mL Blasticidine S HCl (Gibco) were added to keep the stable expression of human FcRn in HMEC1-hFcRn cells^32,41^. The cells were grown in a humid atmosphere of 5% CO_2_ at 37 °C. Madin-Darby Canine Kidney II cells (MDCKII) over-expressing human FcRn (MDCKII-hFcRn) were grown in Dulbecco’s Modified Eagle Medium (DMEM) with GlutaMAX Supplement (Gibco), added 10% FBS, and 300 μg/mL G418. The cells were grown in a humid atmosphere of 5% CO_2_ at 37 °C.

The human erythroblast cell line TF-1 (ATCC, CRL-2003) was cultivated in RPMI-1640 medium (ATCC modification) (Gibco) supplemented with 10% FBS, 25 U/mL PS, and 2 ng/mL recombinant human granulocyte-macrophage colony-stimulating factor (GM-CSF) (PeproTech) for growth. The cells were grown in a humid atmosphere of 5% CO_2_ at 37 °C. The cell lines were not authenticated, but over-expression of human FcRn by the HMEC-1 and MDCK cell lines was routinely monitored using an in-house produced anti-human FcRn antibody by flow cytometry.

### Protein design and production

A baculovirus expression vector system was used to produce soluble truncated His-tagged human FcRn containing its extracellular domains^42–44^. In brief, confluent High Five cells were transferred to Erlenmeyer flasks and cultured in suspension at 1 × 10^6^ cells/mL in an orbital shaker at 27 °C and 160 rpm. A viral stock of baculovirus, kindly gifted by Prof. Sally Ward (University of Southampton, UK), containing a vector encoding a soluble, truncated form of human FcRn was used to transfect the cells (2 mL/L). Following transfection, cells were left in the orbital shaker for 72 h with a temperature adjusted to 23–24 °C, at which point the supernatant was harvested after 10–20 min centrifugation at 2700 × *g*, filtered through a 0.2 μm filter, and adjusted to pH 7.2 using 1x phosphate-buffered saline (PBS) (Sigma-Aldrich) pH 10.9. The His-tagged human FcRn was purified using affinity chromatography on HisTrap HP 5 mL columns (Cytiva), as described by the manufacturer, on a peristaltic pump (Heidolph). Equilibration of the column was done with 1x PBS followed by 25 mM imidazole (Sigma-Aldrich) in PBS (pH 7.2), while 250 mM imidazole in 1x PBS (pH 7.3) was used for elution.

cDNA encoding full-length human serum albumin, including the engineered variants AQ (K500A/H510Q) and QMP (E505Q/T527M/K573P), was expressed in pcDNA3.1(+) or pFUSEss vectors (InvivoGen). To generate vectors encoding EPO-albumin fusions, cDNA encoding human EPO in frame with human serum albumin variants was sub-cloned into the two expression vectors. The restriction sites HindIII/Xhol were used for pcDNA3.1(+), with the EPO leader sequence. For pFUSEss vectors, EPO cDNA was sub-cloned in frame with the N-terminal end of albumin using the restriction sites EcoRI/ClaI, with IL-2 leader sequence.

The pcDNA3.1(+) expression vectors were transiently transfected into HEK293E cells using Polyethylenimine Max (Polysciences), and the pFUSEss expression vectors into either HEK293E cells using Polyethylenimine Max (Polysciences) or Expi293F cells using the ExpiFectamine 293 Transfection Kit (Thermo Fisher Scientific), as described by the manufacturer. The supernatant containing protein was harvested every to every second day for 10–14 days, or as a single harvest at day 4–5, for HEK293E or Expi293F, respectively.

The cells were pelleted by centrifugation for 10–20 min at 2700 × *g*, and the supernatant was run through a 0.2 μm filter before it was purified using a CaptureSelect anti-human serum albumin (Thermo Fisher Scientific) packed column (Repligen), as described by the manufacturer, on a peristaltic pump (Heidolph). Elution of captured protein was done with 0.1 M Glycine-HCl pH 3.0 neutralized with 1 M Tris-HCl pH 8.5 or using 2 M MgCl_2_ pH 7.4. The eluted proteins were concentrated and buffer changed to 1x PBS (Sigma-Aldrich) using Amicon Ultra-15 mL 30 K columns (Merck Millipore) followed by SEC using Superdex 200 Increase 10/300 GL column (Cytiva) coupled to an ÄKTA Avant 25 instrument (Cytiva) with an Alias Autosampler (Agilent).

The monomeric fractions were up-concentrated by Amicon Ultra-4 mL 30 K columns (Merck Millipore) before concentrations were determined using a DS-11+ spectrophotometer (Denovix) and the purity assessed by non-reduced SDS-PAGE using Bolt 12% Bis-Tris Plus Gel (Invitrogen) and Spectra Multicolor Broad Range Protein Ladder (Thermo Fisher Scientific), as described by the manufacturer.

### ELISA

ELISA was used to accompany several of the procedures. In short, the following general protocol was used, with specifics for each analysis described below. The desired protein diluted in PBS was coated onto 96-well EIA/RIA Polystyrene High Bind Microplates (Corning) overnight at 4 °C or 2 h at room temperature. Following a 1-h blocking step at room temperature with 200 μL/well of PBS containing 0.05 % (v/v) Tween 20 (T) (Sigma-Aldrich) and 4% (w/v) skimmed milk powder (M) (Sigma-Aldrich) (PBS/T/M), the wells were washed three consecutive times with 250 μL/well of PBS/T. The samples containing unknown protein levels or known standards were added at 100 μL/well in a titration series and incubated at room temperature for 1–2 h, before the wells were washed as above. The sample was detected using 100 μL/well of ALP-conjugated secondary antibodies incubated for 1 h at room temperature, followed by a wash cycle and then addition of 100 μL/well of p-nitrophenyl phosphate substrate tablets or powder (Sigma-Aldrich) dissolved in diethanolamine buffer (pH 9.8) (Sigma-Aldrich) to a final concentration of 1 mg/mL. A Sunrise spectrophotometer (TECAN) was used to measure the absorbance at 405 nm, with a reference wavelength of 620 nm.

All EPO-albumin fusions produced were tested for binding to albumin through a sandwich albumin ELISA, running a titration series of EPO-albumin fusions or known albumins, between a goat-anti human albumin antibody (Sigma-Aldrich, A1151) (1:2000) as coat and an ALP-conjugated goat-anti human albumin secondary antibody (Bethyl Laboratories, A80-229AP) (1:4000).

Human albumin-levels in plasma, sera, and whole blood were measured as above, except 2 μg/mL monoclonal anti-human albumin antibody (clone 15C7) (Thermo Fisher Scientific, MA1-90420) was used for coating to eliminate cross-binding between the polyclonal anti-human albumin and endogenous mouse serum albumin in blood samples.

EPO-levels in sera and whole blood were measured using a human EPO ELISA kit (Thermo Fisher Scientific, BMS2035-2) according to the manufactures protocol.

The development of anti-drug antibodies against EPO fusions or QMP albumin in the pharmacokinetic studies in Tg32 mice was evaluated by coating plates with 0.5 µg/mL of each respective EPO fusion or unfused QMP albumin. Plasma samples collected on day 27 were added at a final dilution of 1:50 (50 µL/well) alongside a concentration gradient (0.0005–1 µg/mL) of monoclonal anti-human albumin antibody (clone 15C7) (50 µL/well) and pooled reference plasma samples from early time points (5 min for intravenous and 8 h for subcutaneous), as well as naïve serum and a representative positive control. Detection was performed using an ALP-conjugated anti-mouse IgG Fc antibody from goat (Sigma-Aldrich, A2429) (1:5000).

Data points presented were above the LLOD and above the lower limit of quantification (LLOQ). The LLOD for each ELISA run was defined as the mean OD value of blank wells (or those containing naïve serum) + 10 × SD of these, and the LLOQ was defined as the values by which Prism manages to interpolate using the respective standard curves. In cases where the LLOD was above the LLOQ, the values were excluded from the figure presentation and the NCA.

### EPOR binding

The binding kinetics between the generated EPO-albumin fusions and human EPOR were studied using SPR performed on a Biacore T200 instrument (Cytiva). Series S CM5 Sensor chips (Cytiva) were immobilized with His-tagged recombinant human EPOR (Sino Biological) (200 RU) using an Amine Coupling Kit (Cytiva), as described by the manufacturer. A 2-fold dilution series of EPO-albumin fusion or controls (2.5–0.019 nM) in HBS-P+ pH 7.4 (Cytiva) was run over the immobilized surface at 30 μL/min at 25 °C for 210 s, followed by dissociation in HBS-P+ for 960 s, in duplicate. The chip surfaces were regenerated with 2 M MgCl_2_ pH 7.4 with 30 s contact time and 10 s stabilization time between consecutive sample measurements. Binding responses from reference CM5 flow cells and blank injections were subtracted to correct for non-specific binding and bulk refractive index effects using the BIAevaluation software version 4.1 (Cytiva). Kinetic parameters were obtained by globally fitting all concentrations of each titration series, including duplicate injections, to a 1:1 Langmuir binding model, yielding a single set of association (*k*_*a*_) and dissociation (*k*_*d*_) rate constants per experiment with standard errors derived from the covariance matrix of the fit.

Following the same protocol, the binding kinetics between the EPO-albumin fusions and mouse EPOR were determined, with the following alterations: his-tagged recombinant mouse EPOR (Sino Biological) was immobilized (200 RU), and the EPO-albumin fusions or controls were run from 10 to 0.15 nM with 600 s dissociation.

### EPO-dependent proliferation

The proliferative effect of the EPO-albumin fusions and controls was determined utilizing the human erythroblast cell line TF-1. In 48-well plates (Costar), 9 × 10^3^ viable cells were seeded per well in growth medium without GM-CSF, and simultaneously medium containing a gradient of EPO fusion or darbepoetin alfa (final concentration of 100.0–0.0064 nM) was added to the cells at a final volume of 100 μL. Wells with media absent of GM-CSF and EPO were included. Cells were incubated in a humid atmosphere of 5% CO_2_ at 37 °C for 72 h, before 10 μL of CCK-8 reagent (Sigma-Aldrich) were added per well. Following 17 h of incubation, the amount of generated formazan was determined by measuring the absorbance at 450 nm, with a reference wavelength of 620 nm using the Sunrise spectrophotometer. The signal from cells without growth stimuli was used as reference for calculating relative proliferation.

### FcRn binding

The binding between human FcRn and albumin or albumin-fusions was measured by ELISA, with the general protocol as described above. In short, a human IgG1 variant (M252Y/S254T/T256E/H433K/N434F; YTE/KF; Abdeg) (8 μg/mL), with specificity for 4-hydroxy-3-iodo-5-nitrophenylacetic acid)^45^, was used for coating. All steps after blocking were done in buffers adjusted to pH 5.5 or 7.4. The following layers were added subsequent to the washing and incubation steps as above; Glutathione S-transferase-fused soluble recombinant human FcRn (2 μg/mL), 4-fold serial dilution of purified EPO-albumin variants (236.0–0.0009 nM), and ALP-conjugated goat-anti human albumin secondary antibody.

The binding kinetics between the generated EPO-albumin fusions and the human FcRn were studied using SPR, as above. Series S CM5 Sensor chips were immobilized with the EPO-albumin fusions or controls (200 RU) using an Amine Coupling Kit, as described by the manufacturer. In duplicates, a 2-fold dilution series of his-tagged human FcRn (3000–5.86 nM, or 1000–1.95 nM for strong binders) was run over the immobilized surface at 30 μL/min at 25 °C for 120 s, followed by dissociation for 600 s, with phosphate buffer pH 5.5 as running and dilution buffer. The chip surfaces were regenerated with phosphate buffer pH 7.4 with 210 s contact time and 10 s stabilization time between consecutive sample measurements. Binding responses from reference CM5 flow cells and blank injections were subtracted to correct for non-specific binding and bulk refractive index effects using BIAevaluation software version 4.1 (Cytiva). Kinetic parameters were obtained by globally fitting all concentrations of each titration series, including duplicate injections, to a 1:1 Langmuir binding model, yielding a single set of *k*_*a*_ and *k*_*d*_ per experiment with standard errors derived from the covariance matrix of the fit.

Similarly, the binding affinity at neutral pH was measured under the same conditions, but using phosphate buffer pH 7.4 as running, dilution and regeneration buffer. Also, only a duplicate of the highest concentration of human FcRn was injected, and kinetics were not derived.

### Analytical human FcRn affinity chromatography

A human FcRn coupled affinity column (Roche) connected to an ÄKTA Avant 25 instrument was used to study FcRn affinity of the EPO-albumin variants through a pH gradient from 5.5 to 8.8. Briefly, 154 μg of the EPO-albumin variants diluted in 20 mM MES 140 mM NaCl (pH 5.5) was injected onto the column with the same running buffer at pH 5.5. Through the run, a gradual increase in pH occurs due to the mixture of the pH 5.5 buffer with a 20 mM Tris/HCl 140 NaCl (pH 8.8) buffer over 110 min. The pH was monitored by a pH detector (Cytiva) to determine the pH as a function of retention time.

### HERA

HERA^32^ was performed to study FcRn-mediated cellular recycling. In short, 3.75 × 10^4^ viable HMEC-1-hFcRn cells were seeded into 48-well cell culture plates (Costar) and cultured for 1 day. On the day of the experiment, the cells were washed and starved for 1 h in Hank’s balanced salt solution (HBSS) pH 7.4 (Gibco). A total of 800 nM of the EPO-albumin variants or unfused albumin variants in 125 µL HBSS were added per well. Following 3 h of incubation, the buffer was removed from the wells, and the cells were washed with ice cold HBSS before room temperature assay medium (complete cell culture medium without FBS, Blasticidin S HCl, and G418, supplemented with 1x Eagle’s Minimum Essential Medium Non-Essential Amino Acids Solution (Gibco)) was added. After another 3 h of incubation, the media were collected and the cells lysed by addition of ice-cold RIPA lysis buffer (Thermo Fisher) supplemented with 1x Complete Protease Inhibitor (Roche) and a 10-min incubation on ice on a tilting board. Following centrifugation for 15 min at 10,000 × *g* to remove cellular debris, the lysates were transferred to 96-well sample plates and stored at −20 °C. A plate duplicate was lysed after the removal of samples for estimation of uptake into the cells. The amounts of protein in different steps through the assay were quantified using two-way human albumin ELISA.

### Transwell assay

A transcytosis assay^25^ was adapted and performed using MDCKII-hFcRn cells. In short, 1.25 × 10^6^ viable cells were seeded per well, in wells of 0.4 µm Pore PET Membrane Inserts (CellQART) in Costar 12-well TC-treated Multiple Well Plates (Corning) equilibrated overnight with complete cell culture medium. After culturing the cells for 24 h, the cells were washed three times with pre-warmed HBSS and the transepithelial electrical resistance (400–700 Ω × cm^2^) was measured using a Millicell ERS-2 Voltohmmeter (Merck Millipore). Subsequently, the cells were starved in HBSS for 1 h, before 800 nM of the EPO-albumin fusions or controls diluted in 200 µL HBSS were added per well (apical side) in quadruplets. After 4 h of incubation 900 µL of sample were harvested from the basolateral side of each well. The amounts of protein in the sample from the basolateral side were quantified by two-way human albumin ELISA.

### In vivo studies in mice

All mice included in this study were housed under controlled environments, including ventilated cages with regularly change of bedding and nesting material, with free access to water and standard rodent chow, light/dark cycle of 12 h and regulated ambient temperature (21 °C ± 2 °C) and relative humidity (30–70%). The experiments were conducted at either the Jackson Laboratory (JAX Preclinical Services sites Bar Harbor, ME, USA or Alachua, FL, USA) in accordance with approved guidelines and regulations after approval by the Animal Care and Use Committee at the Jackson Laboratory, or at the Section of Comparative Medicine, Rikshospitalet, Oslo University Hospital, Norway, after approval from the Norwegian Food Safety Authority, and performed in accordance with the Guide for the Care and Use of Laboratory Animals of the Norwegian National Institute of Health. The animal facility is classified as specific pathogen-free as defined by the Federation of European Laboratory Animal Science Associations. Experimental and control animals were bred in the same facility and co-housed. Mice were given Purina 5LL4 irradiated diet (the Jackson Laboratory), SDS RM3 irradiated or Ssniff V1154 irradiated diet (Section of Comparative Medicine).

### Pharmacokinetic studies in vivo

Homozygous Tg32 mice (B6.Cg-*Fcgrt*^*tm1Dcr*^Tg(FCGRT)32Dcr/DcrJ; (JAX stock #014565), which express human FcRn and mouse serum albumin, and homozygous Tg32 Alb KO mice (B6.Cg-Tg(FCGRT)32Dcr *Alb*^*em12Mvw*^ *Fcgrt*^*tm1Dcr*^/MvwJ; JAX stock #025201), which express human FcRn and are KO for albumin, were used to determine the plasma half-life of the EPO-albumin variants. For studies conducted in homozygous Tg32 mice, groups of 6 animals (3 females and 3 males) aged 9 weeks weighing 21–25 g were used. Test articles were administered by either intravenous or subcutaneous injection at 2.0 mg/kg (corresponding to 23.6 nmol/kg). Blood samples were collected at 5 min (only post-intravenous injection), 8 h, and on days 1, 2, 3, 5, 7, 9, 12, 15, 18, 21, 24, and 27 post-injection. For studies in the homozygous Tg32 Alb KO mice, gender mixed groups of 5 mice aged 7 weeks weighing 18–27 g were used. Test articles were administered by intravenous injection at 2.0 mg/kg (corresponding to 23.6 nmol/kg). Blood samples were collected at days 1, 2, 3, 4, 5, 7, 10, 12, 16, and 19 post-injection. In both studies, blood samples were collected by retro-orbital bleeding (25 µL). To prevent coagulation of the blood, samples were mixed with 1 µL 1% K3-EDTA immediately after being taken, followed by centrifugation at 17,000 × *g* for 5 min at 4 °C. Plasma from the blood sample was isolated and diluted 1:2 (Tg32) or 1:10 (Tg32 Alb KO) in 50% glycerol/PBS solution and stored at −20 °C until analysis by ELISA. The injections and collections of samples were carried out at the Jackson Laboratory (Tg32 Alb KO mice run at JAX Preclinical Services, Bar Harbor, ME, USA, and Tg32 mice run at JAX Preclinical Services, Alachua, FL, USA), followed by shipment of plasma samples to Rikshospitalet (Oslo, Norway) for analyses. The experiments and procedures used were approved by the Animal Care and Use Committee at The Jackson Laboratory and performed in accordance with the approved guidelines and regulations.

Data from the pharmacokinetic studies are presented as plasma concentration over time and as the percentage remaining in circulation at each time point relative to day 1 (=100%). Pharmacokinetic parameters (AUC, area under the curve from time 0 to the last measured time point; Cmax, maximum concentration following administration; CL, clearance; MRT, mean residency time; *V*ss, volume of distribution at steady state; *T*_1/2_; plasma half-life; R^2^, coefficient of determination) were calculated using NCA including all time points above LLOD and/or LLOQ, and performed using gPKPDSim in MATLAB (version R2022b, MathWorks)^33^.

### Intravenous administration in vivo

Homozygous Tg32 mice (B6.Cg-*Fcgrt*^*tm1Dcr*^Tg(FCGRT)32Dcr/DcrJ; (JAX stock #014565) 14 males aged 16–18 weeks weighing 22.9–30.3 g and 15 females aged 18–19 weeks weighing 23.3–26.8 g were used to study the proliferative effect of the test articles post intravenous administration. In short, each mouse received an injection of 200 µL containing 9.15 µg (0.54 µM) EPO-QMP or equimolar amounts of darbepoetin alfa (4 μg) intravenously in the tail vein. Blood was withdrawn from the saphenous vein at 2, 4, 7, 10, 14, 17, 21, 24, and 30 days post-administration using micro capillary pipettes (Hirschman). The female mice were used for RBC and HCT count and total blood ELISA, while the male mice were used only for ELISA. Sera was isolated by centrifugation at 17,000 × *g* for 15–30 min at 4 °C and stored at −20 °C until analysis, while total blood was collected by immediate mixing in equal amounts of 2 mM EDTA and kept on ice or 4 °C until analysis.

### Mucosal delivery in vivo

Homozygous Tg32 mice (B6.Cg-*Fcgrt*^*tm1Dcr*^Tg(FCGRT)32Dcr/DcrJ; (JAX stock #014565) which express human FcRn and mouse albumin, males aged 10 weeks were used to determine the mucosal uptake and recycling post intranasal delivery of the EPO-albumin variants, and 7–8 weeks males (weighing 24.1–27.3 g) or 23 weeks females (weighing 23.4–31.4 g) for studying the proliferative effect of the test articles. In short, mice were anesthetized intraperitoneally with 5 µL/g of ZRF (250 mg/mL of Zoletil Forte, 20 mg/mL of Rompun, and 50 µg/mL of Fentanyl in saline solution) (Section for Comparative Medicine, Rikshospitalet, Oslo University Hospital) before intranasal delivery of a 10 µL droplet into each nostril containing 80 μg (47.12 µM) EPO-albumin fusion or equimolar amounts of darbepoetin alfa (35 μg) while under sufficient sedation. Blood was withdrawn from the saphenous vein at 0, 9, 24, 48, 72, and 96 h or at 3, 5, 7, 10, 12, 14, 17, 19, and 21 days post-administration using micro capillary pipettes (Hirschman). Sera was isolated by centrifugation at 17,000 × *g* for 15–30 min at 4 °C and stored at −20 °C until analysis, while total blood was collected by immediate mixing in equal amounts of 2 mM EDTA and kept on ice or 4 °C until analysis.

The intravenous and mucosal deliveries in homozygous Tg32 mice were carried out in accordance with the national guidelines and regulations at the Section for Comparative Medicine, Rikshospitalet, Oslo University Hospital (Oslo, Norway), after approval from the Norwegian Food Safety Authority.

### Proliferation in vivo post mucosal and intravenous delivery

Analyses on whole blood mixed in 2 mM EDTA (ratio 1:2) were performed less than 1 h after blood withdrawal using an ABX Micros 60 Hematology Analyzer with internal software version 1.7.4 (Horiba Medical), and each sample was vortexed immediately prior to analysis.

### Data analysis, graphing, and illustration

Chromatography data were obtained using Äkta Avant 25 UNICORN (Version 10.7, Cytiva) and further processed using Microsoft Excel. ELISA data were processed by Microsoft Excel. SPR data were obtained and processed using Biacore T200 Evaluation Software (Version 3.0, Cytiva). In vivo pharmacokinetic analyses (NCA) were obtained using gPKPDSim (Version 1.1.3) running within SimBiology inside MATLAB (version R2022b, MathWorks). Blood parameters for the proliferation study were obtained using ABX Micros 60 Hematology Analyzer with internal software version 1.7.4 (Horiba Medical), and Microsoft Excel was used for data processing. Data were analyzed and presented using GraphPad Prism for Mac (Version 8.1.2 or newer; GraphPad Software), which was also used for statistical analyses and figure assembly. Biorender.com was used to create illustrations under an academic license plan.

### Reporting summary

Further information on research design is available in the Nature Portfolio Reporting Summary linked to this article.

## Supplementary information

Supplementary Information

Reporting Summary

Transparent Peer Review file

## Source data

Source Data

Source Data 2

## Data Availability

All data are included in the manuscripts, Supplementary Information, Source Data file, or available from the authors, as are unique reagents used in this Article. The raw numbers for charts and graphs are available in the Source Data file whenever possible. Source data are provided with this paper.
