# Supplementary material for: A human erythropoietin-albumin fusion protein tailored for enhanced plasma half-life and delivery across mucosal surfaces
Source: Nat Commun. 2026 Oct 28;17:10780. doi: 10.1038/s41467-026-78243-z (PMC13716905; doi:10.1038/s41467-026-78243-z)
Supplement: Supplementary file 1 [file 41467_2026_78243_MOESM1_ESM.pdf]

Supplementary Information

A human erythropoietin-albumin fusion protein tailored for enhanced plasma half-life and delivery across mucosal surfaces

Aina Karen Anthi[1,2,3], Daniela Bejan[1,2,3], Simone Mester[1,2,3], Nijaguna Bethur[4], Elena Gonzalo-Gil[5], Sopisa Benjakul[1,2,3], Kristin Hovden Aaen[1,2,3], Tilman Schlothauer[6], Inger Sandlie[7] and Jan Terje Andersen[1,2,3]*.

[1]Precision Immunotherapy Alliance (PRIMA), University of Oslo; 0372 Oslo, Norway.

[2]Department of Immunology, Oslo University Hospital Rikshospitalet; 0372 Oslo, Norway.

[3]Institute of Clinical Medicine and Department of Pharmacology, University of Oslo and Oslo University Hospital Rikshospitalet; 0372 Oslo, Norway.

[4]The Jackson Laboratory, Bar Harbor, ME 04609, USA.

[5]The Jackson Laboratory, Alachua, FL 32615, USA.

[6]Biochemical and Analytical Research, Large Molecule Research, Roche Pharma Research and Early Development (pRED), Roche Innovation Center Munich; 82377 Penzberg, Germany.

[7]Department of Biosciences, University of Oslo; 0371 Oslo, Norway.

*Corresponding author

Jan Terje Andersen, Department of Immunology, Oslo University Hospital Rikshospitalet and University of Oslo, Norway, PO Box 4950, N-0424 Oslo, Norway.

E-mail: j.t.andersen@medisin.uio.no

Supplementary Information List:

Supplementary Figure 1: EPO-QMP produced in HEK293E and Expi293F are equally functional.

Supplementary Figure 2: Sequence alignment of human and mouse EPOR.

Supplementary Figure 3: Binding kinetics between mouse EPOR and EPO-albumin fusions.

Supplementary Figure 4: Distinct human FcRn binding properties and in vivo characteristics of the engineered EPO-albumin fusions.

Supplementary Figure 5: EPO-QMP and darbepoetin alfa levels in sera post intravenous and intranasal delivery to mice.

Supplementary Table 1: SPR-derived binding kinetics between EPO-albumin fusions and mouse EPOR.

Supplementary Table 2: Overview of statistical methods and p-values.

Supplementary Table 3: Summary of statistical analyses from in vivo proliferation.

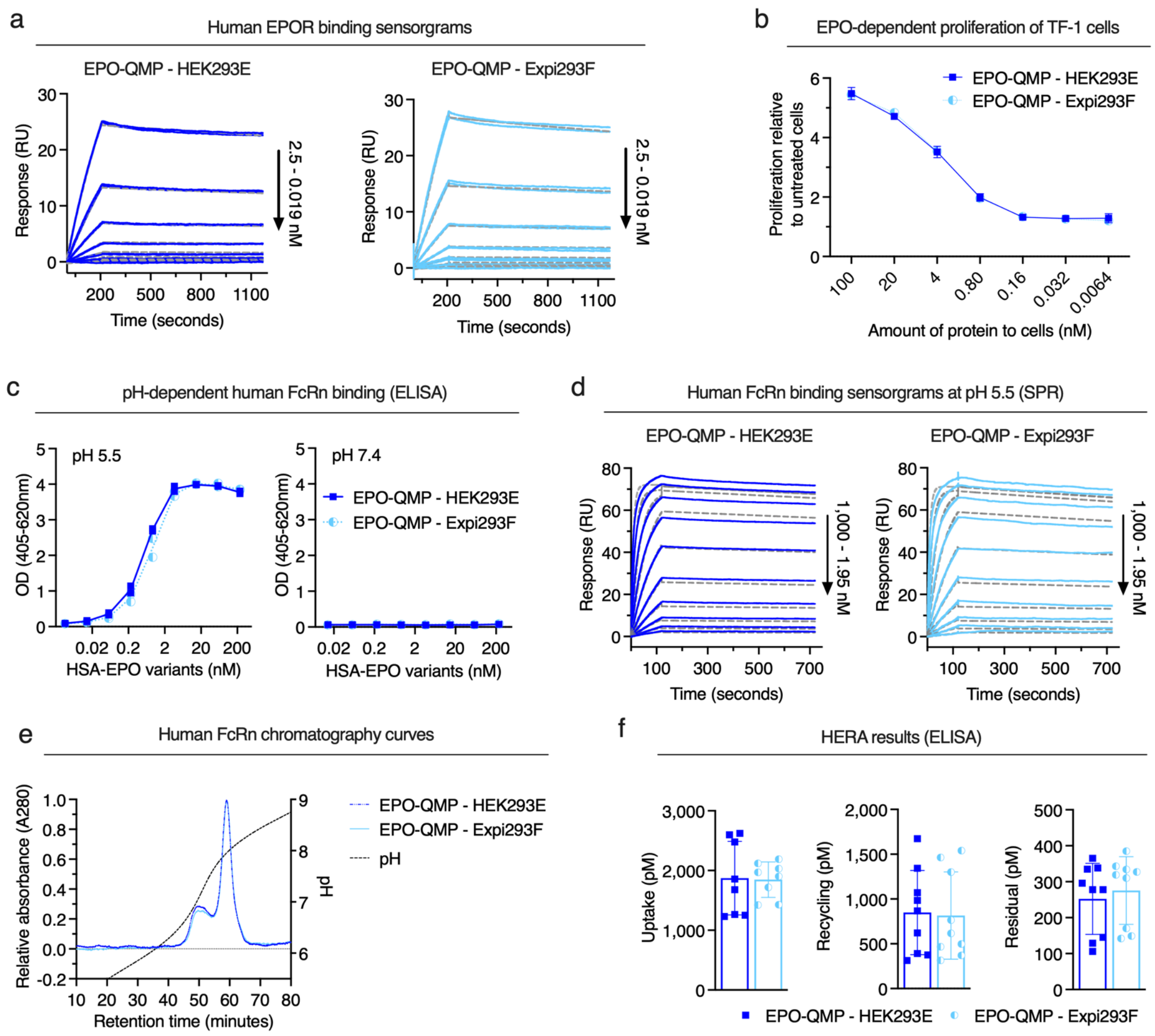

Supplementary Figure 1: EPO-QMP produced in HEK293E and Expi293F are equally functional. a SPR binding curves of 2-fold serial dilutions (2.5-0.019 nM) of EPO-QMP injected in duplicates over a CM5 chip immobilized with human EPOR (~ 200 RU). **b** The relative cell proliferation values for human TF-1 cells exposed to serial titrations (100-0.0064 nM) of EPO-QMP (mean of $n = 4 \pm$ SD), followed by adding CCK-8. **c** ELISA binding curves between EPO-QMP and human FcRn at pH 5.5 (each replicate of duplicates) and pH 7.4 (singlets), using the ELISA setup with a human IgG1 variant (YTE/KF) as coat and detecting with ALP-conjugated anti-human albumin. **d** SPR sensorgrams obtained at pH 5.5 following injection of 2-fold serial titrations of human FcRn (1,000-1.95 nM) in duplicates over a CM5 chip immobilized with EPO-QMP (~ 200 RU). **e** The binding and release profiles of EPO-QMP (154 µg injected) as relative absorbance and as a function of the pH gradient, using analytical human FcRn chromatography. **f** Results from a two-way human albumin ELISA setup,

quantifying albumin levels in harvested HERA samples (800 nM injected) of 3 individual experiments run with triplicates (mean ± SD). Binding curves in **a** and **d** presented as each replicate of duplicates in solid lines, and curves fitted to the 1:1 Langmuir binding model are presented as dashed lines. Source data are provided as a Source Data file.

```
sp|P19235|EPOR_HUMAN      APPPNLPDPKFESKAALLAARGPEELLCFTERLEDLVCFWEEAASAGVGPGNYSFSYQLE  60
sp|P14753|EPOR_MOUSE      APSPSLPDPKFESKAALLASRGSEELLCFTQRLEDLVCFWEEAASSGMD-FNYSFSYQLE  59
                          ** *.**************:** *******:**************:*:.  *********

sp|P19235|EPOR_HUMAN      DEPWKLCRLHQAPTARGAVRFWCSLPTADTSSFVPLELRVTAASGAPRYHRVIHINEVVL  120
sp|P14753|EPOR_MOUSE      GESRKSCSLHQAPTVRGSVRFWCSLPTADTSSFVPLELQVTEASGSPRYHRIIHINEVVL  119
                          .*  * * ******.**:********************:** ***:*****:********

sp|P19235|EPOR_HUMAN      LDAPVGLVARLADESGHVVLRWLPPPETPMTSHIRYEVDVSAGNGAGSVQRVEILEGRTE  180
sp|P14753|EPOR_MOUSE      LDAPAGLLARRAEEGSHVVLRWLPPPGAPMTTHIRYEVDVSAGNRAGGTQRVEVLEGRTE  179
                          ****.**:** *:*..********** :***:************ **..****:******

sp|P19235|EPOR_HUMAN      CVLSNLRGRTRYTFAVRARMAEPSFGGFWSAWSEPVSLLTPSDLDPLILTLSLILVVILV  240
sp|P14753|EPOR_MOUSE      CVLSNLRGGTRYTFAVRARMAEPSFSGFWSAWSEPASLLTASDLDPLILTLSLILVLISL  239
                          ******** ****************.*********.**** ***************:* :

sp|P19235|EPOR_HUMAN      LLTVLALLSHRRALKQKIWPGIPSPESEFEGLFTTHKGNFQLWLYQNDGCLWWSPCTPFT  300
sp|P14753|EPOR_MOUSE      LLTVLALLSHRRTLQQKIWPGIPSPESEFEGLFTTHKGNFQLWLLQRDGCLWWSPGSSFP  299
                          ************:*:***************************** *.******** : *

sp|P19235|EPOR_HUMAN      EDPPASLEVLSERCWGTMQAVEPGTDDEGPLLEPVGSEHAQDTYLVLDKWLLPRNPPSED  360
sp|P14753|EPOR_MOUSE      EDPPAHLEVLSEPRWAVTQAGDPGADDEGPLLEPVGSEHAQDTYLVLDKWLLPRTPCSEN  359
                          ***** ******  *.. ** :**:*****************************.* **:

sp|P19235|EPOR_HUMAN      LPGPGGSVDIVAMDEGSEASSCSSALASKPSPEGASAASFEYTILDPSSQLLRPWTLCPE  420
sp|P14753|EPOR_MOUSE      LSGPGGSVDPVTMDEASETSSCPSDLASKPRPEGTSPSSFEYTILDPSSQLLCPRALPPE  419
                          * ******* *:***.**:*** * ***** ***:* :************** * :* **

sp|P19235|EPOR_HUMAN      LPPTPPHLKYLYLVVSDSGISTDYSSGDSQGAQGGLSDGPYSNPYENSLIPAAEPLPPSY  480
sp|P14753|EPOR_MOUSE      LPPTPPHLKYLYLVVSDSGISTDYSSGGSQGVHGDSSDGPYSHPYENSLVPDSEPLHPGY  479
                          ***************************.***.:*. ******:******:* :*** *.*

sp|P19235|EPOR_HUMAN      VACS  484
sp|P14753|EPOR_MOUSE      VACS  483
                          ****
```

Supplementary Figure 2: Sequence alignment of human and mouse EPOR. Sequence alignment displaying amino acid similarity indicated by asterisks between the human and mouse EPO receptor (excluding signal peptide). Sequence identity match: 82.19%. Made by using Clustal Omega (1.2.4) multiple sequence alignment with the following Uniprot accession numbers: Human - P19235, mouse - P14753.

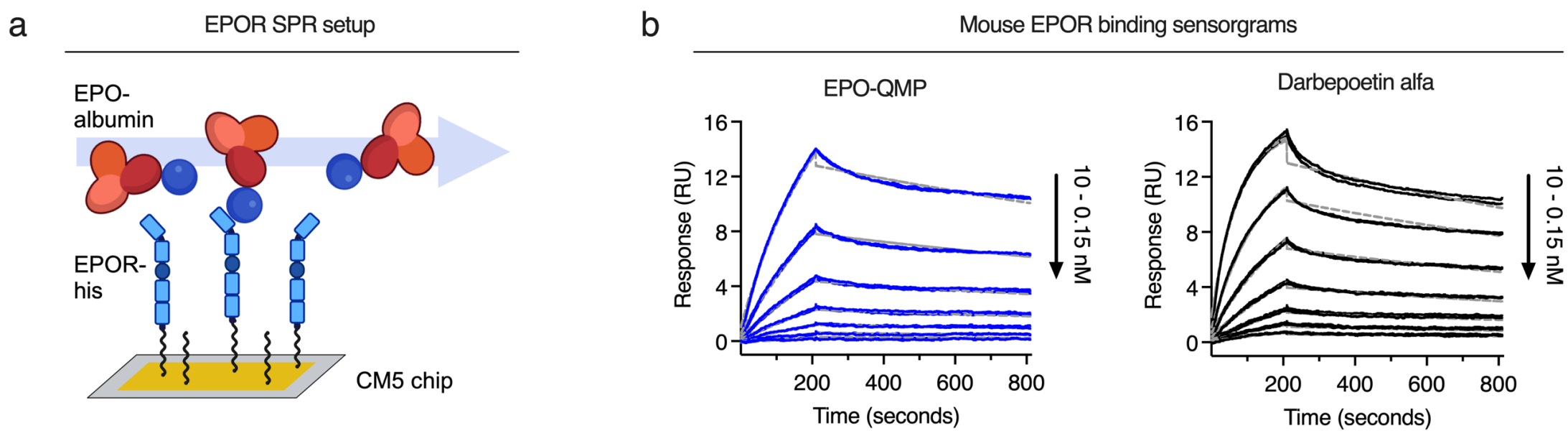

Supplementary Figure 3: Binding kinetics between mouse EPOR and EPO-albumin fusions. a Illustration of the SPR setup for measuring the binding kinetics between the mouse EPOR and EPO-albumin where the EPOR was covalent immobilized by amine coupling to a CM5 chip followed by injection of monomeric EPO-albumin fusions or darbepoetin alfa. **b** The resulting binding curves of 2-fold serial dilutions (10-0.15 nM) of EPO-QMP and darbepoetin alfa injected in duplicates over immobilized mouse EPOR (~ 200 RU), where each replicate is presented as a solid line. Curves fitted to the 1:1 Langmuir binding model are presented as dashed lines. **a** created in BioRender. Anthi, A. (2026) https://BioRender.com/7faobuw. Source data are provided as a Source Data file.

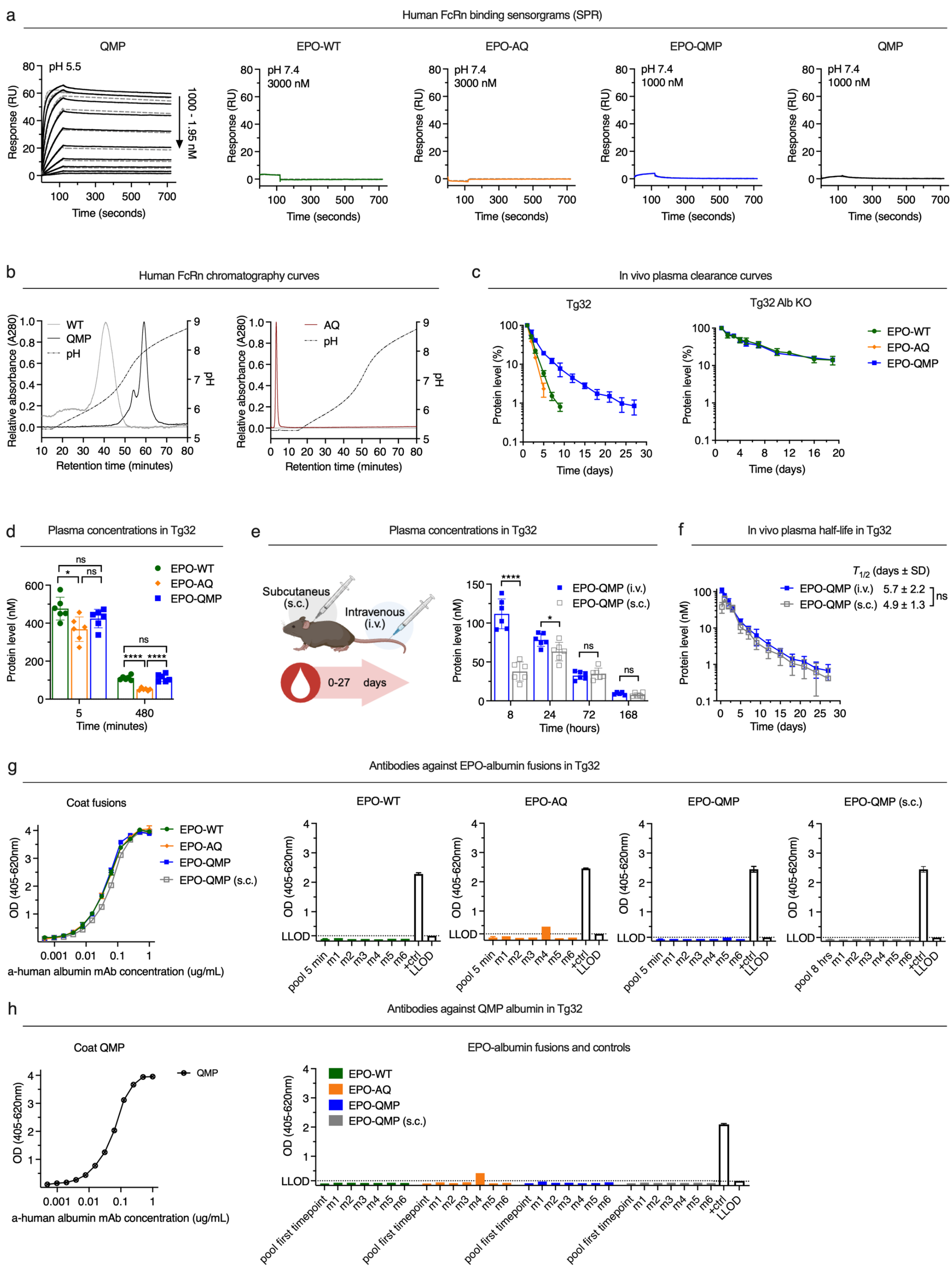

Supplementary Figure 4: Distinct human FcRn binding properties and in vivo characteristics of the engineered EPO-albumin fusions. **a** SPR sensorgrams obtained at pH 5.5 following injection of 2-fold serial titrations (1,000-1.95 nM) or at pH 7.4 following a single concentration (3,000 nM for EPO-WT and EPO-AQ, and 1,000 nM for EPO-QMP) of human FcRn over a CM5 chip immobilized with EPO-albumin fusion or unfused albumin QMP (~

200 RU). Binding curves presented as each replicate of duplicates (at pH 5.5) or singlet (pH 7.4) as solid lines and curves fitted to the 1:1 Langmuir binding model are presented as dashed lines. **b** The binding and release profiles of unfused human albumin variants (154 µg injected) as relative absorbance and as a function of the pH gradient, using analytical human FcRn chromatography. **c** The in vivo plasma clearance curves post intravenous administration of 2.0 mg/kg EPO-albumin fusion proteins in Tg32 mice *n* = 6 (3M/3F) and in Tg32 Alb KO mice *n* = 5, presented as group mean ± SD of the percentage remaining compared to D1. **d** Protein levels of EPO-albumin fusions in plasma from Tg32 mice following intravenous (i.v.) delivery, shown as dotted bar plots with group mean ± SD and single data points. **e** Illustration of study in Tg32 mice where EPO-QMP (2 mg/kg) was given by either i.v. or subcutaneous (s.c) delivery to *n* = 6 (3M/3F) mice. The protein levels in plasma at time points 8-186 hours are shown as dotted bar plots with group mean ± SD and single data points. **f** Plasma clearance of EPO-QMP in Tg32 mice following i.v or s.c. delivery, shown as group mean ± SD of *n* = 6 (3M/3F). Group average plasma half-life values ($T_{1/2}$ ± SD) are shown in days**.** Protein concentrations in plasma were quantified using a two-way anti-albumin ELISA, and show all values interpolated (Sigmoidal, 4PL, X is concentration) using Prism that were above the LLOD (all other values are defined as not detected). Statistical details and exact *p*-values are listed in Supplementary Table 2. ****$p \leq 0.0001$; ***$p \leq 0.0010$; **$p \leq 0.0100$; *$p \leq 0.0500$; ns $p > 0.0500$. **g-h** Anti-drug antibodies studied in plasma samples from Tg32 mice on day 27, detected using a polyclonal anti-mouse IgG antibody on samples applied to ELISA plates coated with **g** each representative EPO-albumin fusion or **h** unfused QMP albumin. Each standard curve run in duplicate is presented as the mean OD value ± SD. The OD value of each sample is shown in separate graphs with representative positive control and LLOD from the respective plates. **e** partially created in BioRender. Anthi, A. (2026) https://BioRender.com/7faobuw. Source data are provided as a Source Data file.

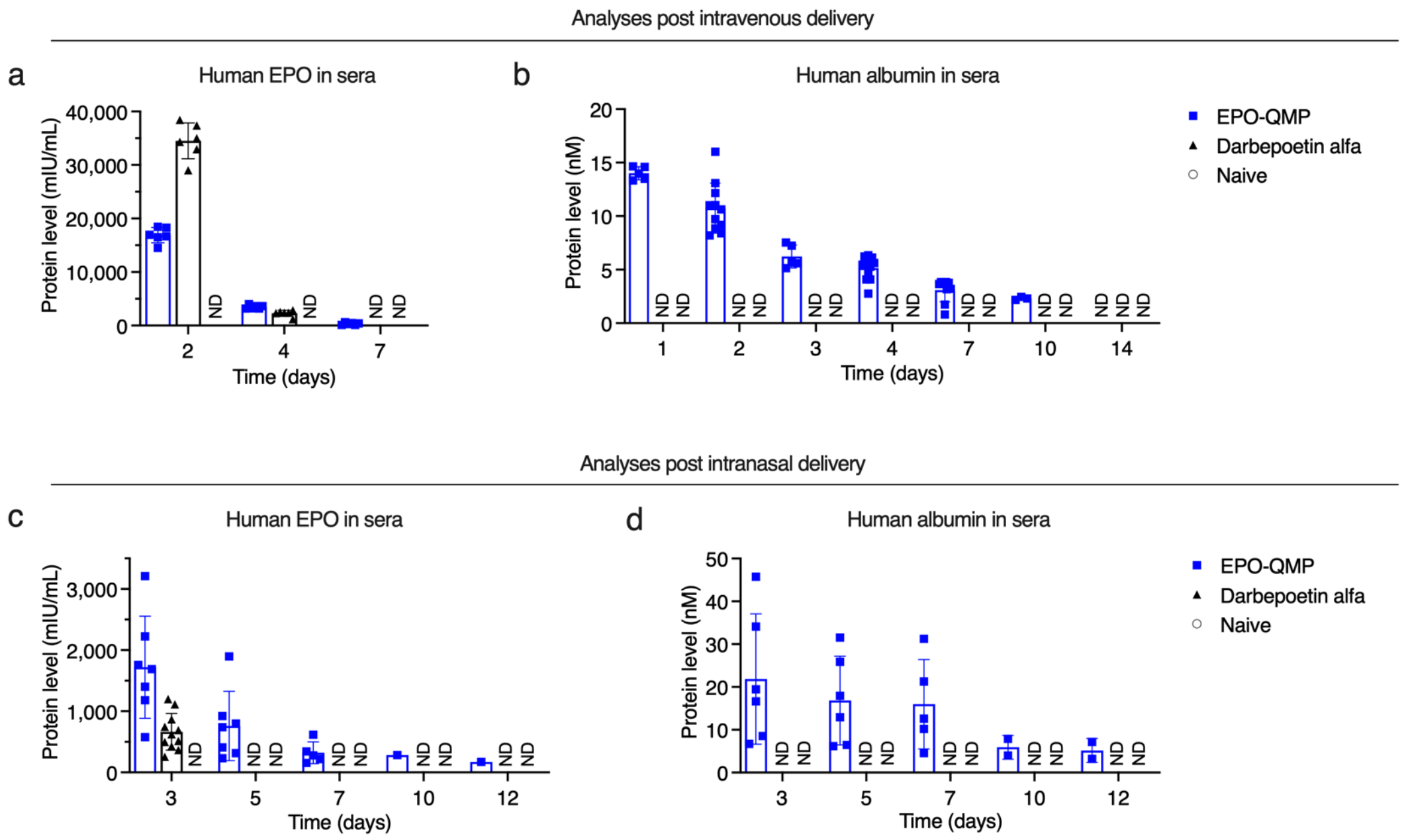

Supplementary Figure 5: EPO-QMP and darbepoetin alfa levels in sera post intravenous and intranasal delivery to mice. Results from ELISA analyses of sera after (**a-b**) intravenous delivery (9 µg EPO-QMP or 4 µg darbepoetin alfa) or (**c-d**) intranasal delivery (80 µg EPO-QMP or 35 µg darbepoetin alfa), quantified using (**a** and **c**) human EPO-specific or (**b** and **d**) human albumin-specific ELISAs. ELISA data presented as individual samples with mean ± SD for each time point (**a** *n* = 6, 6, 2, all male (one experiment), **b** *n* = 11 (6M/5F), 11 (6M/5F), 4 (2M/2F) (two experiments), **c** *n* = 7 (3M/4F), 13 (5M/8F), 4 (2M/2F) (two experiments), **d** *n* = 7 (3M/4F), 13 (5M/8F), 5 (3M/2F) (two experiments) for EPO-QMP, darbepoetin alfa and naive, respectively), and shows all values interpolated (Sigmoidal, 4PL, X is concentration) using Prism that were above the LLOD (all other values are defined as not detected; ND). Source data are provided as a Source Data file.

Supplementary Table 1: SPR-derived binding kinetics between EPO-albumin fusions and mouse EPOR. Kinetic parameters were obtained by global fitting of all concentrations, including duplicate injections, using a 1:1 Langmuir binding model. Standard errors (SE) are derived from the covariance matrix of the fit.

	**Mouse EPOR**			
**Variant**	**k_a (10^4 M^{-1} s^{-1})**	**k_d (10^{-5} s^{-1})**	**KD (10^{-9} M)**	**Chi2**
EPO-QMP	44.000 ± 0.113	40.100 ± 0.074	0.911	0.027
Darbepoetin alfa	130.000 ± 0.076	48.200 ± 0.074	0.369	0.054

Supplementary Table 2: Overview of statistical methods and exact *p*-values.

Figure	Statistical test	Sample	*p*-value
4c	One-way ANOVA with Tukey's multiple comparison (two-tailed)	EPO-WT vs EPO-AQ	>0.9999
		EPO-WT vs EPO-QMP	<0.0001
		EPO-WT vs WT	0.9681
		EPO-WT vs QMP	<0.0001
		EPO-AQ vs EPO-QMP	<0.0001
		EPO-AQ vs WT	0.9810
		EPO-AQ vs QMP	<0.0001
		EPO-QMP vs WT	<0.0001
		EPO-QMP vs QMP	<0.0001
		WT vs QMP	<0.0001
4d	One-way ANOVA with Tukey's multiple comparison (two-tailed)	EPO-WT vs EPO-AQ	0.9999
		EPO-WT vs EPO-QMP	<0.0001
		EPO-WT vs WT	0.9994
		EPO-WT vs QMP	<0.0001
		EPO-AQ vs EPO-QMP	<0.0001
		EPO-AQ vs WT	0.9954
		EPO-AQ vs QMP	<0.0001
		EPO-QMP vs WT	<0.0001
		EPO-QMP vs QMP	>0.9999
		WT vs QMP	<0.0001
4e	One-way ANOVA with Tukey's multiple comparison (two-tailed)	EPO-WT vs EPO-AQ	0.9987
		EPO-WT vs EPO-QMP	<0.0001
		EPO-WT vs WT	0.0355
		EPO-WT vs QMP	<0.0001
		EPO-AQ vs EPO-QMP	<0.0001
		EPO-AQ vs WT	0.0288
		EPO-AQ vs QMP	<0.0001
		EPO-QMP vs WT	0.0002
		EPO-QMP vs QMP	0.9018
		WT vs QMP	<0.0001
4g	One-way ANOVA with Tukey's multiple comparison (two-tailed)	EPO-WT vs EPO-AQ, Day 1	<0.0001
		EPO-WT vs EPO-QMP, Day 1	0.0001
		EPO-AQ vs EPO-QMP, Day 1	<0.0001
		EPO-WT vs EPO-AQ, Day 3	0.0003
		EPO-WT vs EPO-QMP, Day 3	<0.0001
		EPO-AQ vs EPO-QMP, Day 3	<0.0001
		EPO-WT vs EPO-AQ, Day 5	0.0215
		EPO-WT vs EPO-QMP, Day 5	<0.0001
		EPO-AQ vs EPO-QMP, Day 5	<0.0001
4h	Unpaired t-test (two-tailed)	EPO-WT vs EPO-AQ	<0.0001
		EPO-WT vs EPO-QMP	0.0011
		EPO-AQ vs EPO-QMP	0.0002
4i	One-way ANOVA with Tukey's	EPO-WT vs EPO-AQ, Day 1	0.0001
		EPO-WT vs EPO-QMP, Day 1	0.9616

	multiple comparison (two-tailed)	EPO-AQ vs EPO-QMP, Day 1	<0.0001
	Unpaired t-test (two-tailed)	EPO-WT vs EPO-QMP, Day 3	0.8278
		EPO-WT vs EPO-QMP, Day 5	0.0842
4j	Unpaired t-test (two-tailed)	EPO-WT vs EPO-QMP	0.7944
5b	One-way ANOVA with Tukey's multiple comparison (two-tailed)	EPO-WT vs EPO-AQ	0.9996
		EPO-WT vs EPO-QMP	0.0006
		EPO-WT vs WT	0.9330
		EPO-WT vs AQ	0.9963
		EPO-WT vs QMP	<0.0001
		EPO-AQ vs EPO-QMP	0.0003
		EPO-AQ vs WT	0.8134
		EPO-AQ vs AQ	0.9670
		EPO-AQ vs QMP	<0.0001
		EPO-QMP vs WT	0.0065
		EPO-QMP vs AQ	0.0020
		EPO-QMP vs QMP	<0.0001
		WT vs AQ	0.9973
		WT vs QMP	<0.0001
		AQ vs QMP	<0.0001
5d	Unpaired t-test (two-tailed)	EPO-WT vs EPO-QMP	0.0023
6b-c		See Supplementary Table 3	
6d	Unpaired t-test (two-tailed)	EPO-QMP vs Darbepoetin alfa, Day 2	<0.0001
		EPO-QMP vs Darbepoetin alfa, Day 4	0.0023
6f-g		See Supplementary Table 3	
6h	Unpaired t-test (two-tailed)	EPO-QMP vs Darbepoetin alfa, Day 3	0.0060
Supplementary Figure 4d	One-way ANOVA with Tukey's multiple comparison (two-tailed)	EPO-WT vs EPO-AQ, 5 minutes	0.0146
		EPO-WT vs EPO-QMP, 5 minutes	0.2933
		EPO-AQ vs EPO-QMP, 5 minutes	0.2479
		EPO-WT vs EPO-AQ, 8 hours	<0.0001
		EPO-WT vs EPO-QMP, 8 hours	0.9982
		EPO-AQ vs EPO-QMP, 8 hours	<0.0001
Supplementary Figure 4e	Unpaired t-test (two-tailed)	Intravenous vs subcutaneous, 8 hours	<0.0001
		Intravenous vs subcutaneous, 24 hours	0.0287
		Intravenous vs subcutaneous, 72 hours	0.5428
		Intravenous vs subcutaneous, 168 hours	0.2230
Supplementary Figure 4f	Unpaired t-test (two-tailed)	EPO-QMP vs EPO-WT	0.4504

Supplementary Table 3: Summary of statistical analyses from in vivo proliferation.

Figure	Statistical test	Timepoint	*p*-values for given sample comparison		
			EPO-QMP vs darbepoetin alfa	EPO-QMP vs naive	Darbepoetin alfa vs naive
6b	One-way ANOVA with Tukey's multiple comparison (two-tailed)	Day 7	0.9837	<0.0001	<00001
		Day 10	0.3946	<00001	0.0002
		Day 14	0.0048	<00001	0.0335
		Day 17	0.0019	<00001	0.2046
		Day 21	0.0119	0.0018	0.1199
		Day 24	0.0012	0.0004	0.1345
		Day 30	0.1048	0.0096	0.1413
6c	One-way ANOVA with Tukey's multiple comparison (two-tailed)	Day 10	0.8507	<00001	<00001
		Day 14	0.0301	<00001	0.0161
		Day 17	0.007	<00001	0.144
		Day 21	0.0864	0.0249	0.3825
		Day 24	0.015	0.0152	0.6765
		Day 30	0.8542	0.3681	0.5837
6f	One-way ANOVA with Tukey's multiple comparison (two-tailed)	Day 3	0.9911	0.9991	0.9975
		Day 5	0.9801	0.0088	0.0055
		Day 7	0.0164	<0.0001	0.0021
		Day 10	0.0147	0.0013	0.1855
		Day 12	0.1654	0.0423	0.4652
		Day 14	0.044	0.0045	0.2275
		Day 17	0.0593	0.0207	0.5322
		Day 19	0.051	0.0061	0.2596
		Day 21	0.3299	0.0958	0.5036
6g	One-way ANOVA with Tukey's multiple comparison (two-tailed)	Day 3	0.9232	0.9602	0.7987
		Day 5	0.8922	0.0047	0.0007
		Day 7	0.1507	0.0004	0.0074
		Day 10	0.0614	0.0053	0.2046
		Day 12	0.6568	0.3061	0.6502
		Day 14	0.3073	0.0497	0.3304
		Day 17	0.2551	0.115	0.6613
		Day 19	0.0279	0.0067	0.3989
		Day 21	0.9796	0.9278	0.8304
