# Supplementary material for: A human erythropoietin-albumin fusion protein tailored for enhanced plasma half-life and delivery across mucosal surfaces
Source: Nat Commun. 2026 Oct 28;17:10780. doi: 10.1038/s41467-026-78243-z (PMC13716905; doi:10.1038/s41467-026-78243-z)
Supplement: Supplementary file 2 [file 41467_2026_78243_MOESM2_ESM.pdf]

nature portfolio

Corresponding author(s): Jan Terje Andersen

Last updated by author(s): Jul 10, 2026

Reporting Summary

Nature Portfolio wishes to improve the reproducibility of the work that we publish. This form provides structure for consistency and transparency in reporting. For further information on Nature Portfolio policies, see our Editorial Policies and the Editorial Policy Checklist.

Statistics

For all statistical analyses, confirm that the following items are present in the figure legend, table legend, main text, or Methods section.

n/a	Confirmed	
☐	☒	The exact sample size (*n*) for each experimental group/condition, given as a discrete number and unit of measurement
☐	☒	A statement on whether measurements were taken from distinct samples or whether the same sample was measured repeatedly
☐	☒	The statistical test(s) used AND whether they are one- or two-sided<br>*Only common tests should be described solely by name; describe more complex techniques in the Methods section.*
☒	☐	A description of all covariates tested
☐	☒	A description of any assumptions or corrections, such as tests of normality and adjustment for multiple comparisons
☐	☒	A full description of the statistical parameters including central tendency (e.g. means) or other basic estimates (e.g. regression coefficient) AND variation (e.g. standard deviation) or associated estimates of uncertainty (e.g. confidence intervals)
☐	☒	For null hypothesis testing, the test statistic (e.g. *F*, *t*, *r*) with confidence intervals, effect sizes, degrees of freedom and *P* value noted<br>*Give P values as exact values whenever suitable.*
☒	☐	For Bayesian analysis, information on the choice of priors and Markov chain Monte Carlo settings
☐	☒	For hierarchical and complex designs, identification of the appropriate level for tests and full reporting of outcomes
☒	☐	Estimates of effect sizes (e.g. Cohen's *d*, Pearson's *r*), indicating how they were calculated

Our web collection on statistics for biologists contains articles on many of the points above.

Software and code

Policy information about availability of computer code

Data collection	- Microsoft Excel; version 16.16.19 (200210) or newer<br>- Basic Local Alignment Search Tool (Nucleotide BLAST and Protein BLAST); https://blast.ncbi.nlm.nih.gov/Blast.cgi<br>- EMBOSS Needle Pairwise Alignment Tool; https://www.ebi.ac.uk/Tools/psa/emboss_needle/<br>- ClustalW2; https://www.ebi.ac.uk/Tools/msa/clustalw2/ (retired)<br>- Expasy Translate; https://web.expasy.org/translate/<br>- Expasy ProtParam tool; https://web.expasy.org/protparam/<br>- Magellan; Tecan Biosciences; version 7.2<br>- Unicorn; Cytiva; version 10.7<br>- Biacore T200 Evaluation Software; Cytiva; version 3.0<br>- ABX Micros 60 Hematology Analyzer with software version 1.7.4; Horiba Medical
Data analysis	- Microsoft Excel; version 16.16.19 (200210) or newer<br>- GraphPad Prism 10 software for Mac; version 10.4.1<br>- Unicorn; Cytiva; version 10.7<br>- Biacore T200 Evaluation Software; Cytiva; version 3.0<br>- gPKPDsim Toolbox; Mathworks; Version 1.1.3 (doi: 10.1007/s10928-017-9562-9)<br>- SimBiology; Mathworks; Version R2022b

For manuscripts utilizing custom algorithms or software that are central to the research but not yet described in published literature, software must be made available to editors and reviewers. We strongly encourage code deposition in a community repository (e.g. GitHub). See the Nature Portfolio guidelines for submitting code & software for further information.

Data

Policy information about availability of data

All manuscripts must include a data availability statement. This statement should provide the following information, where applicable:

- Accession codes, unique identifiers, or web links for publicly available datasets
- A description of any restrictions on data availability
- For clinical datasets or third party data, please ensure that the statement adheres to our policy

All data are included in the manuscripts, Supplementary Information, Source Data file or available from the authors, as are unique reagents used in this Article. The raw numbers for charts and graphs are available in the Source Data file whenever possible. Source data are provided with this paper.

Research involving human participants, their data, or biological material

Policy information about studies with human participants or human data. See also policy information about sex, gender (identity/presentation), and sexual orientation and race, ethnicity and racism.

Reporting on sex and gender	Not applicable, the study did not involve human participants, their data, or biological material.
Reporting on race, ethnicity, or other socially relevant groupings	Not applicable, the study did not involve human participants, their data, or biological material.
Population characteristics	Not applicable, the study did not involve human participants, their data, or biological material.
Recruitment	Not applicable, the study did not involve human participants, their data, or biological material.
Ethics oversight	Not applicable, the study did not involve human participants, their data, or biological material.

Note that full information on the approval of the study protocol must also be provided in the manuscript.

Field-specific reporting

Please select the one below that is the best fit for your research. If you are not sure, read the appropriate sections before making your selection.

☒ Life sciences ☐ Behavioural & social sciences ☐ Ecological, evolutionary & environmental sciences

For a reference copy of the document with all sections, see nature.com/documents/nr-reporting-summary-flat.pdf

Life sciences study design

All studies must disclose on these points even when the disclosure is negative.

Sample size

Sample size is in this setting relevant to mainly 1) animal experiments and 2) cellular experiments.

Sample size in animal experiments was determined on the basis of ethical perspectives (the three Rs) and previously published data (see list 1) in the transgenic mouse strains, confirming the sufficiency of the used sample size for statistical interpretation.

Sample size in cellular experiments was determined on the basis of previously published data (see list 2) regarding method variability, the inclusion of technical replicates across biological replicates and necessary sample size for statistical interpretation where indicated.

List 1) Animal experiments: PMID: 35982144, PMID: 34109608, PMID: 32313072, PMID: 35118359, PMID: 15331566, PMID: 2070021, PMID: 41022699.
List 2) Cellular experiments: PMID: 29434196, PMID: 35982144, PMID: 37827516, PMID: 41022699.

Data exclusions

Data was excluded from further interpretation if found to be below the sensitivity of the relevant analytic method, and has no impact on the inferred conclusions where applicable.

Data points presented were above the lower limit of detection (LLOD) and above the lower limit of quantification (LLOQ). The LLOD for each ELISA run was defined as the mean OD value of blank wells (or those containing naïve serum) + 10 × SD of these, and the LLOQ was defined as the values by which Prism manages to interpolate using the respective standard curves. In cases where the LLOD was above the LLOQ, the values were excluded from the figure presentation and the NCA.

Replication

To ensure reproducibility of experimental findings, experiments were performed with at least two (biochemical) or three (cellular) technical replicates of each individual data point, and independent assays were run. Due to ethical considerations, animal experiments were performed only once. For analyses of blood/plasma tests were performed twice when sufficient sample volume.
All stated hardware routinely underwent maintenance and quality assessment both prior to and during the data collection period.
All attempts at replication were successful.

Randomization	Samples were, where feasible, randomized in terms of location on plate/assay. Animals were placed into groups at a random basis, with some deviation for the experiment including male mice. In that case mice that were already housed together were used as one group. Cages were given a random position in the housing racks.
Blinding	Investigators were partially blinded during in vitro and in vivo analyses. We saw no difference between blinded and non-blinded experiments/ analyses.

Reporting for specific materials, systems and methods

We require information from authors about some types of materials, experimental systems and methods used in many studies. Here, indicate whether each material, system or method listed is relevant to your study. If you are not sure if a list item applies to your research, read the appropriate section before selecting a response.

Materials & experimental systems

n/a	Involved in the study
☐	☒ Antibodies
☐	☒ Eukaryotic cell lines
☒	☐ Palaeontology and archaeology
☐	☒ Animals and other organisms
☒	☐ Clinical data
☒	☐ Dual use research of concern
☒	☐ Plants

Methods

n/a	Involved in the study
☒	☐ ChIP-seq
☒	☐ Flow cytometry
☒	☐ MRI-based neuroimaging

Antibodies

Antibodies used

Commercial antibodies used in experimental analysis (Tradename; Supplier; Reference; Lot.nr; RRID):
1) Anti-human albumin produced in goat; Sigma-Aldrich; A1151; SLCF5233; AB_257921.
Dilution 1:2000.
2) ALP-conjugated anti-human albumin produced in goat; Bethyl Laboratories; A80-229AP; 10; AB_67020.
Dilution 1:4000.
3) Human Serum Albumin Monoclonal Antibody (15C7); Thermo Fisher Scientific; MA1-90420; YE3901834; AB_1958507.
Working concentration 2ug/mL.
4) Anti-Mouse IgG (Fc specific)-Alkaline Phosphatase antibody produced in goat; Sigma; A2429; 029M4801V; AB_258000.
Dilution 1:5000

Antibodies from kits:
5) Human EPO ELISA kit; Thermo Fisher Scientific; BMS2035-2; 387840-004 & 406029-007; AB_2575339.

Antibodies produced in-house used in experimental analysis:
6) NIP-specific human IgG1 with M252Y/S254T/T256E/H433K/N434F (MST/HN) amino acid substitutions (doi: 10.4049/jimmunol.1401218; doi: 10.1038/nbt1143). Working concentration: 8ug/mL.

Validation

All antibodies (commercial and in-house) are widely used in the laboratory and are regularly tested against in-house samples of known effect. Any abnormal results are detected, tracked and reported (to manufacturer if necessary). In addition, Citeab.com is used to check the commercial antibodies, and the CoA of commercial antibodies are checked upon delivery.

Eukaryotic cell lines

Policy information about cell lines and Sex and Gender in Research

Cell line source(s)

Cell lines:
- High Five cells, Invitrogen, B85502.
- HEK293E, ATCC, CRL-1573.
- Expi293F, Gibco, A14527.
- HMEC-1-HA-FcRn-EGFP: Boston Children’s Hospital, Harvard Medical School and Harvard Digestive Diseases Center, USA; generation of described in doi: 10.1091/mbc.E13-04-0174.
- MDCKII-hFcRn: pRED Roche Innovation Center Munich, Germany.
- TF-1, ATCC, CRL-2003.

Authentication

Cell lines not authenticated, but the hFcRn expression of the hFcRn overexpressing cell lines were routinely monitored by flow cytometry.

Mycoplasma contamination

All cell lines tested negative for mycoplasma contamination.

Commonly misidentified lines (See ICLAC register)

Not applicable.

Animals and other research organisms

Policy information about studies involving animals; ARRIVE guidelines recommended for reporting animal research, and Sex and Gender in Research

Laboratory animals	The following mice at specified ages were used in the study:<br><br>- Homozygous Tg32 mice (B6.Cg-Fcgrttm1DcrTg(FCGRT)32Dcr/DcrJ; (JAX stock #014565). For studies with IV delivery, age 9 weeks (half-life study) or 16-18 weeks (proliferation study). For the study with SC delivery, age 9 weeks. For studies with mucosal delivery, age 10 weeks (mucosal delivery study), and 7-8 or 23 weeks (proliferation study).<br><br>- Homozygous Tg32 Alb KO mice (B6.Cg-Tg(FCGRT)32Dcr Albem12Mvw Fcgrttm1Dcr/MvwJ; JAX stock #025201), age 7 weeks (half-life study).
Wild animals	The study did not involve wild animals.
Reporting on sex	Sex is reported in the Methods section describing each in vivo animal study. Both female and male mice were used, and findings apply to both.
Field-collected samples	The study did not involve field-collected samples.
Ethics oversight	The animal studies were carried out at The Department for Comparative Medicine, Oslo University Hospital (Rikshospitalet, Oslo, Norway) in accordance with the Guide for the Care and Use of Laboratory Animals of the Norwegian National Institute of Health, and the experiments were approved by The Norwegian Food Safety Authority, or at The Jackson Laboratory (JAX Services, Bar Harbor, MA, USA) in accordance with IACUC approved procedures.

Note that full information on the approval of the study protocol must also be provided in the manuscript.

Plants

Seed stocks	Not applicable, the study did not involve plants.
Novel plant genotypes	Not applicable, the study did not involve plants.
Authentication	Not applicable, the study did not involve plants.
