# Supplementary material for: A human erythropoietin-albumin fusion protein tailored for enhanced plasma half-life and delivery across mucosal surfaces
Source: Nat Commun. 2026 Oct 28;17:10780. doi: 10.1038/s41467-026-78243-z (PMC13716905; doi:10.1038/s41467-026-78243-z)
Supplement: Supplementary file 3 [file 41467_2026_78243_MOESM3_ESM.pdf]

nature portfolio

Peer Review File

A human erythropoietin-albumin fusion protein tailored for enhanced plasma half-life and delivery across mucosal surfaces

Corresponding Author: Professor Jan Terje Andersen

This file contains all reviewer reports in order by version, followed by all author rebuttals in order by version.

Version 0:

Reviewer comments:

Reviewer #1

(Remarks to the Author)
The objective of this manuscript is to provide pre-clinical evidence for potential therapeutic use of fusion protein human erythropoietin-albumin (EPO-QMP) for nasal delivery. The major results include demonstration that FcRn-mediated recycling of human albumin in transgenic mice extends EPO-QMP half-life and EPO-QMP is efficacious after both intravenous and intranasal deliveries. The manuscript is well written, and results delivered in a clear manner supported by extensive figures and tables. Major concerns include non-compartmental analysis of in vivo data including calculation of half-lives. Other concerns are explained below.

Major concerns:

1) Problematic analysis of pharmacokinetic (PK) data. Figure 4 and Table 3 contain results of analysis of EPO constructs and albumin in Tg32 and Tg32AlbKO mice following intravenous dose (1.8 mg/kg, 2 mg/kg) injections via the tail vain. Key mechanisms of EPO elimination are binding to EPOR and non-specific clearance presumably by EPO uptake by endothelial cells followed by intracellular degradation. Authors recognize importance of EPOR mediated clearance in Discussion (l. 350-353). EPOR is critical at EPO plasma concentrations at which EPORs are not saturated that are in the range of endogenous EPO plasma concentrations in mouse (10-100 pM). This is the EPO concentration range that would qualify to be called the terminal phase, and this is part of PK data that should be used to determine “the terminal plasma half-life”(Table 3). The ranges of EPO-construct concentrations studied are well above EPOR saturation levels. This means that data shown in Fig. 4 represents non-specific elimination or tissue distribution phase of PK profile. Authors should demonstrate the former. Please provide the full PK data starting from day 1 until the limit of quantification is reached (that for EPO-QMP is way beyond day 10) to show there is no biexponential pattern. Also, the data show in Fig. 4 should be consistent with the data used for non-compartmental analysis results of which are shown in Table 3. The half-lives (T1/2) from this table should be consistent with T1/2beta vales reported in Fig. 4 and elsewhere in the manuscript. Otherwise, T1/2beta might be well describing the distributional clearance. Finally, missing EPO measurements between 0 and 24 h can substantially contribute to AUC calculations (and subsequently CL). This should be addressed in Discussion.

2) Selection of darbepoetin alfa (DARB) as a reference EPO construct. In Introduction (l. 54) authors present DARB as an EPO-construct with an extended plasma half-life compared to EPO (epogen). Further DARB is used as a reference to compare with in vivo efficacy of EPO-QMP. The comparisons are in favor of EPO-QMP (Fig. 6). Authors conclude that extended half-life of EPO-QMP is a cause of improved efficacy over DARB (l. 368-370). This conclusion is correct, but it creates impression that the half-life extension by linking EPO to albumin is a major therapeutic advantage (l. 39-40). The therapeutic impact of EPO-QMP is not its better in vivo efficacy than DARB but its ability to deliver EPO intranasally. Authors should discuss why they did not choose for a reference another long-lasting ESA, such as continuous erythropoietin receptor activator (CERA, methoxy polyethylene glycol-epoetin beta) with a longer half-life than DARB and better efficacy. There are other long-lasting ESAs outperforming DARB on a 1:1 molar dosing that could have been selected for a reference. Fig. 6 would not have shown such a favorable RBC response. While having an external reference is applaudable, picking DARB is questionable since it hints marketing EPO-QMP.

Minor concerns

3) l. 39 “should” is too strong. Please consider “could/might”.
4) l. 56. Please add references for other long-lasting ESAs (see 2)).
5) l. 165. What is a soluble form of the FcRn receptor?
6) l. 482-483. Please provide limits of quantification for EPO ELISAs. Include these limits in Fig. 4.
7) l. 620. Why did blood sampling not last until day 30, as for IV delivery?
8) l. 630. Normalizing EPO PK profiles by day 1 measurement complicates analysis. Please consider using actual data in Fig. 4 as in Supplementary Figure 4 C but in semi-log scale (see 1))
9) l. 637-639. Specify what data were used for NCA analysis by gPKPDsim. These data should be presented in Fig. 4 or in Supplementary Materials. Describe how gPKPDsim selects terminal phase of PK data for T1/2 calculation. State if this is consistent with Fig. 4.
10) Table 1. Please provide standard errors of estimates for Ka and Kd. Units for Ka are wrong.
11) Table 3. Please use molar units for NCA parameters AUC, Cmax to enable comparison with binding parameters from Table 1 and HERA results from Fig. 4. Units for Dose should be ug (provide molar equivalent). All parameters should be reported as mean +/- SD of N=5 animals.
10)
11) Fig 2. Please keep the same range for Y axes of plots in Fig. 2B. Please reverse order of X axis in Fig. 2D.
12) Fig. 4H and 4J. Please include full EPO concentration time courses until the limit of quantification is reached. Do not use normalize data. Use molar concentrations for comparison with other results (EPOR and FcRn binding). Thie figure should clarify what data is used for calculation terminal half-life and NCA analysis (see 1)).
13) Fig. 6D and 6E. Please use molar concentrations to enable comparison with other results. X label for Fig. 6E should read “intravenous”. Correct label for Fig. 6H.
14) Supplementary Figure 3 is too small.
15) Supplementary Figure 5. Please use molar concentration units.
16) Supplementary Table 1. Please see 10)

Reviewer #2

(Remarks to the Author)
The manuscript, "A human erythropoietin-1 albumin fusion protein..." is notably well-written with excellent experimental detail provided to support the major claims of increased half-life and improved absorption of the EPO-QMP fusion protein. The authors provide insight into the specific applications of the EPO-QMP and to the utility of the approach for other fusion proteins. I have two recommendations:
1. Provide a summary of the limitations of the study, including the lack of evaluating PKPD from subcutaneous injections, which is a major route of administration for EPO and EPO-derived therapeutics.
2. Modified proteins have the potential to be immunogenic. Although a complete study into immunogenecity is arguably beyond the scope of this report, any guidance or relevant references in this regard would be useful.

Version 1:

Reviewer comments:

Reviewer #1

(Remarks to the Author)
Thank you for addressing my concerns. I do not have any further questions. One suggestion
1) Since you have measured plasma concentrations for i.v. and s.c. administration it will be helpful to report bioavailability either in Table 3 or in Supplementary Figure 4.

Reviewer #2

(Remarks to the Author)
The authors have made substantial, thoughtful changes to respond to previous reviewers’ concerns. While these are mostly sufficient, there are two issues that could be addressed to improve the manuscript further:

1. The authors acknowledge they still cannot define a true terminal half-life at physiologically relevant EPO concentrations (10-100 pM), yet the manuscript continues to report "plasma half-life". The authors report that when they attempted dosing at 2 µg/kg (to get into the 10-100 pM) the fusion protein could not be detected above background at any timepoint. In addition, there is a new statement noting that "the plasma elimination curve for EPO-QMP in the Tg32 mice display several phases, indicating multiple modes of clearance mechanisms"; however, it is not entirely clear if the latter phase reflects a EPOR-mediated elimination only (or other non-specific clearance only), or if there is an additional terminal phase at physiologic relevant concentrations below 100 pM. Although this limitation is honestly disclosed, the half-life differences are central to the conclusions of the manuscript and authors should carefully consider revising their claim of “plasma half-life” to “apparent terminal half-life” or something else more transparent.

2. The justification for using DARB as a reference is better explained, but somewhat side-steps the major concern that the comparison to DARB only shows that modification of EPO with QMP improves half-life over an unmodified form of EPO, but does not demonstrate equivalence, or superiority, over other half-life-extending forms of EPO.

Open Access This Peer Review File is licensed under a Creative Commons Attribution 4.0 International License, which permits use, sharing, adaptation, distribution and reproduction in any medium or format, as long as you give appropriate credit to the original author(s) and the source, provide a link to the Creative Commons license, and indicate if changes were made.
In cases where reviewers are anonymous, credit should be given to 'Anonymous Referee' and the source.
The images or other third party material in this Peer Review File are included in the article's Creative Commons license, unless indicated otherwise in a credit line to the material. If material is not included in the article's Creative Commons license and your intended use is not permitted by statutory regulation or exceeds the permitted use, you will need to obtain permission directly from the copyright holder.
To view a copy of this license, visit https://creativecommons.org/licenses/by/4.0/

Manuscript: NCOMMS-25-22339

Response to reviewers

Reviewer #1 (Remarks to the Author):

The objective of this manuscript is to provide pre-clinical evidence for potential therapeutic use of fusion protein human erythropoietin-albumin (EPO-QMP) for nasal delivery. The major results include demonstration that FcRn-mediated recycling of human albumin in transgenic mice extends EPO-QMP half-life and EPO-QMP is efficacious after both intravenous and intranasal deliveries. The manuscript is well written, and results delivered in a clear manner supported by extensive figures and tables. Major concerns include non-compartmental analysis of in vivo data including calculation of half-lives. Other concerns are explained below.

Major concerns:

1) Problematic analysis of pharmacokinetic (PK) data. Figure 4 and Table 3 contain results of analysis of EPO constructs and albumin in Tg32 and Tg32AlbKO mice following intravenous dose (1.8 mg/kg, 2 mg/kg) injections via the tail vain.

Key mechanisms of EPO elimination are binding to EPOR and non-specific clearance presumably by EPO uptake by endothelial cells followed by intracellular degradation. Authors recognize importance of EPOR mediated clearance in Discussion (l. 350-353). EPOR is critical at EPO plasma concentrations at which EPORs are not saturated that are in the range of endogenous EPO plasma concentrations in mouse (10-100 pM). This is the EPO concentration range that would qualify to be called the terminal phase, and this is part of PK data that should be used to determine "the terminal plasma half-life" (Table 3). The ranges of EPO-construct concentrations studied are well above EPOR saturation levels. This means that data shown in Fig. 4 represents non-specific elimination or tissue distribution phase of PK profile. Authors should demonstrate the former.

Please provide the full PK data starting from day 1 until the limit of quantification is reached (that for EPO-QMP is way beyond day 10) to show there is no biexponential pattern. Also, the data show in Fig. 4 should be consistent with the data used for non-compartmental analysis results of which are shown in Table 3. The half-lives (T1/2) from this table should be consistent with T1/2beta vales reported in Fig. 4 and elsewhere in the manuscript. Otherwise, T1/2beta might be well describing the distributional clearance. Finally, missing EPO measurements between 0 and 24 h can substantially contribute to AUC calculations (and subsequently CL). This should be addressed in Discussion.

We thank the reviewer for the attention towards the pharmacokinetics (PKs) analysis.

We fully agree that the original dataset may not have captured the complete PK profile of EPO-QMP in the human FcRn expressing mice. Therefore, we have repeated the experiment in the Tg32 mice where we intravenously dosed the mice with 2 mg/kg of the different protein designs followed by collection of blood from 5 minutes post injection and up to 30 days. The results showed that we could detect the QMP-containing EPO-fusion protein for 27 days, while the EPO-WT fusion was detected for up to 10 days. This resulted in a 5-fold extended plasma half-life for EPO-QMP compared with that of the WT fusion counterpart, 5.7 vs 1.1 days, respectively (Figure 4h).

Regarding the methods used for PK analysis, we initially employed both Microsoft Excel and the gPKPDSim software in MatLab. To avoid any confusion by the use of two different methods, we

decided to use the non-compartmental analysis (NCA) derived from the gPKPDSim software for analyses included in the revised manuscript.

Furthermore, we agree with the reviewer that the intravenous dose used results in plasma concentrations of EPO-QMP and related constructs that are above the EPOR saturation threshold. As such, the PK profiles presented in the original manuscript reflect not only EPOR-mediated elimination, but also non-specific clearance mechanisms as well as FcRn-mediated rescue from intracellular degradation, depending on the receptor binding kinetics of the three different human albumin variants that are explored as a carrier for EPO. Thus, the use of the term *terminal* plasma half-life is not appropriate. We have revised the manuscript and omitted using the word *terminal*. Throughout the main text, we consistently refer to "plasma half-life", a term encompassing biodistribution, elimination, and clearance from circulation. In Table 3, however, we initially reported the output obtained from gPKPDSim as "Terminal plasma half-life", as described in Hosseini et al., *J. Pharmacokinet. Pharmacodyn.* (2018). We have now revised this for consistency to read "plasma half-life" (highlighted in yellow in Table 3). We have also replaced "T1/2β" with "T1/2" in all figures. All datapoints used for the NCA in Table 3 are presented in Figure 4h and j, and vice versa.

As such, the plasma half-life values given in Figure 4h-j have been updated with results from the repeated PK study in Tg32 mice (pages 10-11, lines 225-248):

"The results from the study in mice expressing human FcRn (Tg32) revealed that similar amounts were detected of EPO-WT and EPO-QMP at both 5 minutes and 8 hours post injection, with a 4-fold drop in amounts between the timepoints (Supplementary Figure 4d). At 24 hours, the amounts of EPO-WT and EPO-QMP dropped further by 8.7- and 5.4-fold, respectively, compared to that measured at 5 minutes. Over time, higher amounts of EPO-QMP were detected than the WT counterpart, with 1.4-fold more at 24 hours, 2.7-fold on day 3, and 4.8-fold on day 5 post injection (Figure 4g-h). On day 5, EPO-QMP was detected at 28-fold lower amounts than after 5 minutes, while the difference for EPO-WT was as much as 150-fold (Figure 4g and Supplementary Figure 4d). The EPO-AQ variant, engineered for reduced FcRn binding, was detected in similar amounts as the other EPO fusions at 5 minutes but decreased by 7.1-fold at 8 hours and as much as 700-fold over the following 5 days, after which the variant was not detected (Figure 4g-h and Supplementary Figure 4d). Impressively, EPO-QMP could be detected for up to 27 days post administration while EPO-WT levels were not detectable after 10 days. As such, the results demonstrated a profound effect of albumin engineering for improved and reduced FcRn engagement.

To derive the plasma half-life values and pharmacokinetic parameters, a non-compartmental analysis (NCA) was performed on the measured plasma concentrations using the gPKPDsim for MatLab [33]. The results showed the slowest clearance for EPO-QMP (60.3 ± 9.3 mL/d/kg), followed by a 1.6-fold faster clearance for the WT counterpart (97.4 ± 6.1 mL/d/kg) (Table 3), while EPO-AQ was cleared rapidly (193.9 ± 30.0 mL/d/kg) (Table 3). The plasma half-life of the QMP-containing variant (5.7 ± 2.2 days) was 5.2-fold longer than that of EPO-WT (1.1 ± 0.1 days) (Figure 4h, Supplementary Figure 4c, and Table 3)."

The paragraph "Half-life calculation" (lines 629-639) in the original manuscript has been removed from Methods as it describes the use of Excel for the calculation of half-life values. Instead, information regarding data presentation and NCA have been added under the "Half-life determination in vivo" section of Methods (page 30, lines 699-706):

Removed (lines 629-639, original manuscript):
"**Half-life calculation**
The half-life data is presented as percentage of the plasma concentration of the EPO-albumin fusion variants remaining in circulation at different time points post injection, compared to day 1. To fit a

straight line through the data, nonlinear regression analysis was performed by using Prism 8, and the plasma half-life was calculated using the formula: $t1/2\beta = \log 0.5/(\log Ae/A0) \times t$, where $t1/2\beta$ is the plasma half-life of the EPO-albumin fusion variant evaluated, *Ae* is the amount of the EPO-albumin fusion remaining, *A0* is the amount of EPO-albumin fusion variant on day 1 and *t* is the elapsed time. gPKPDsim for MatLab was used for non-compartmental pharmacokinetic analysis using the plasma concentration of the fusions post intravenous delivery in human FcRn transgenic mice[29]."

Added (page 30, lines 699-706):

"Data from the half-life studies are presented as plasma concentration over time and as the percentage remaining in circulation at each time point relative to day 1 (= 100 %). Pharmacokinetic parameters (AUC, area under the curve from time 0 to the last measured time point; Cmax, maximum concentration following administration; CL, clearance; MRT, mean residency time; Vss, volume of distribution at steady state; T1/2; plasma half-life; R2, coefficient of determination) were calculated using non-compartmental analysis (NCA) including all time points from until the lower limit of quantification was reached, and performed using gPKPDSim in MATLAB (version R2022b, MathWorks)[33]."

Next, we aimed to detect EPO-QMP at the range of 10-100 pM. This was explored by giving 2 µg/kg to Tg32 mice followed by collection of blood 5 minutes, 6, 12 and 24 hours, and 3, 5, 7, 9, 12, 15, 18, 21, 24, and 27 days post-injection. Unfortunately, even without pre-dilution of plasma samples in glycerol and using low dilutions in the ELISA, the test articles could not be detected at levels above the background signal at any time point. As a result, we could not calculate a terminal half-life when the protein levels were in the range of only 10-100 pM. With this in mind, we have added a statement to the manuscript to bring attention to the multiple modes of clearance of EPO-based drugs (page 18, lines 397-399):

"Of note, the plasma elimination curve for EPO-QMP in the Tg32 mice (Figure 4h) display several phases, indicating multiple modes of clearance mechanisms."

In addition to the study above, we also addressed how subcutaneous delivery would affect plasma half-life. To directly compare with intravenous delivery, a group of mice were given 2 mg/kg of EPO-QMP subcutaneously. This experiment is reported in the revised manuscript as Supplementary Figure 4e-f, and showed peak plasma levels after 24 hours followed by a clearance curve similar to that upon intravenous administration. This resulted in a similar long plasma half-life as that of intravenous delivery with a non-statistically different (5.7 ± 2.2 days versus 4.9 ± 1.3 days).

The comparison between intravenous and subcutaneous delivery has been included in the revised manuscript as follows; Results (pages 11-12, lines 250-263), Discussion (page 18, lines 400-404), Table 3 and Supplementary Figure 4e-f.

Pages 11-12, lines 250-263:

"Furthermore, we addressed how a subcutaneous delivery route would affect the plasma half-life and pharmacokinetics of EPO-QMP with the same dose as before (2.0 mg/kg). While the amounts detected after intravenous delivery decreased 1.4-fold between 8 hours and 24 hours, specifically from 111.8 to 78.4 nM, subcutaneous delivery resulted in a 1.7-fold increase, from 37.7 to 63.3 nM in the same interval (Supplementary Figure 4e). For the timepoints following 24 hours, comparable reductions in detected EPO-QMP were observed for both administration routes (Supplementary Figure 4e-f). Mice given EPO-QMP by the subcutaneous route showed a maximum plasma concentration (Cmax) of 63.3 nM, 6.7-fold lower than that measured after intravenous delivery (Table 3). In addition, the time point at which Cmax was measured was shifted, from 5 minutes with

intravenous delivery to 24 hours with subcutaneous delivery (Supplementary Figure 4d-f). Despite the differences in amounts detected at early time points, the plasma half-life after subcutaneous delivery (4.9 ± 1.3 days) was not statistically different from that after intravenous delivery (Table 3, Supplementary Figure 4f, Supplementary Table 2)."

Page 18, lines 400-404:

"A common route of administration for protein-based therapeutics, including those that are EPO-based, is by subcutaneous delivery. As such, we compared intravenous and subcutaneous administration of EPO-QMP, which resulted in similar plasma elimination curves and derived plasma half-life values, supporting both delivery strategies as relevant for future studies."

Moreover, we agree with the reviewer about the necessity to have measurements taken between 0 and 24 hours to provide a more correct estimation of the AUC and, this way, an improved estimation of total drug exposure over time. In the new in vivo study performed in Tg32 mice, we collected blood at 5 minutes and 8 hours after administration, in addition to the already included 24-hour blood sample. NCA analysis was performed on all datapoints from the new dataset and is now reported in the updated Table 3. Specifically, inclusion of these early time points resulted in higher AUC values compared to the initial study, which correspondingly led to lower clearance values (CL = Dose/AUC). The updated results provide a more accurate characterization of systemic exposure. For the study in Tg32 AlbKO mice where no datapoints are available before 24 hours, we have highlighted this limitation in the discussion (page 18, lines 414-416):

"Notably, caution must be taken when directly comparing the pharmacokinetic measurements derived from the two studies, as the initial phase prior to 24 hours was not included in the Tg32 AlbKO study design."

Notably, in the ELISA performed with plasma samples collected from the albumin deficient Tg32 strain (AlbKO), a higher background signal was measured that resulted in a high lower limit of detection (LLOD) (defined as mean OD value from blank wells + 10 × SD of these blanks), which disabled calculation of the levels of EPO-QMP and EPO-WT at time points after day 10. Repeating the ELISA with limited sample volumes yielded the same results, for reasons unknown. Nevertheless, the generated measurements were used for reanalysis and resulted in detectable levels in plasma up to day 19 for EPO-WT and EPO-QMP, and day 1 for EPO-AQ. Details on how data points were included are described in the answer of comment 6.

2) Selection of darbepoetin alfa (DARB) as a reference EPO construct. In Introduction (l. 54) authors present DARB as an EPO-construct with an extended plasma half-life compared to EPO (epogen). Further DARB is used as a reference to compare with in vivo efficacy of EPO-QMP. The comparisons are in favor of EPO-QMP (Fig. 6). Authors conclude that extended half-life of EPO-QMP is a cause of improved efficacy over DARB (l. 368-370). This conclusion is correct, but it creates impression that the half-life extension by linking EPO to albumin is a major therapeutic advantage (l. 39-40). The therapeutic impact of EPO-QMP is not its better in vivo efficacy than DARB but its ability to deliver EPO intranasally. Authors should discuss why they did not choose for a reference another long-lasting ESA, such as continuous erythropoietin receptor activator (CERA, methoxy polyethylene glycol-epoetin beta) with a longer half-life than DARB and better efficacy. There are other long-lasting ESAs outperforming DARB on a 1:1 molar dosing that could have been selected for a reference. Fig. 6 would not have shown such a favorable RBC response. While having an external reference is applaudable, picking DARB is questionable since it hints marketing EPO-QMP.

We thank the reviewer for the attention towards the choice of reference recombinant EPO. Our criteria for choice were that the reference should be a marketed product containing a human EPO protein sequence and be a widely used erythropoietin stimulator across markets, with a focus on indications requiring long-term treatment. Both methoxy PEG-epoetin beta (Mircera) and darbepoetin alfa (Aranesp) were relevant options, where methoxy PEG-epoetin beta is approved with a further reduced dosing regimen compared to darbepoetin alfa, as stated by the reviewer. Nevertheless, darbepoetin alfa was chosen as its binding affinity towards the hEPOR is closer to that of recombinant human EPO with its 4-fold reduction (3), compared with methoxy PEG-epoetin beta that demonstrates a 50-100-fold reduction in binding affinity (4). We have updated the manuscript with a statement regarding these aspects (Introduction, page 3, lines 46-59, and Discussion, page 16, lines 348-358 and page 19, lines 438-441):

Introduction, page 3, lines 46-59:

"However, the first marketed recombinant EPO products have a rather short plasma half-life ranging from 4-11 hours after intravenous administration in humans, which increases to about a day following subcutaneous administration [6]. To solve this, engineering strategies have been explored to extend the plasma half-life of recombinant EPO. For instance, darbepoetin alfa (Aranesp, Amgen) has five amino acid substitutions that introduce two additional N-glycan sites, compared to the three N- and one O-glycan sites on EPO, extending the plasma half-life in humans from 8.5 to 26.3 hours after intravenous administration [2,7,8]. Another example is methoxy polyethylene glycol (PEG)-epoetin beta (Mircera, Roche) that was designed by chemically conjugating a large PEG moiety (> 30 kDa) site-specifically to recombinant EPO, resulting in a plasma half-life of 134 hours after intravenous administration in anemic patients with chronic kidney disease on dialysis [9–11].

An alternative approach could be to fuse EPO to a protein carrier that can provide prolonged plasma half-life and transcellular transport properties."

Discussion, page 16, lines 348-358:

"Recombinant human EPO exhibits a short plasma half-life of 4-11 hours after intravenous administration in humans [6], which has motivated engineering strategies to extend its systemic exposure, such as to reduce plasma clearance through increased molecular mass by introduction of N-glycan sites as for darbepoetin alfa [2,8] or by chemical conjugation to PEG as for methoxy PEG-epoetin beta [9]. Upon intravenous administration, these strategies prolong their plasma half-lives by approximately 3-fold (25.3 hours) for darbepoetin alfa and 14-fold (134 hours) for methoxy PEG-epoetin beta compared to unmodified recombinant human EPO, allowing for reduced dosing frequency [8,9,11].
An alternative approach to prolong the plasma half-life of recombinant EPO is to genetically fuse it to an FcRn ligand, which will allow receptor-mediated rescue from lysosomal degradation."

Discussion, page 19, lines 438-441:

"Indeed, EPO-QMP exhibited greater erythrocyte proliferation compared with darbepoetin alfa, consistent with the studies on intravenous administration. Of note, a crucial aspect for efficacy is systemic exposure over time and thus the half-life is key. Further studies are needed to compare the efficacy of EPO-QMP with other ESAs."

Please note that the following references were added to support the above:

6. Deicher & Hörl, *Drugs* (2004)
9. Macdougall et al., *Clin. J. Am. Soc. Nephrol.* (2006)

10. Macdougall & Eckardt, *Lancet* (2006)
11. Ohashi et al., *Int. J. Nephrol. Renov. Dis.* (2012)

Regarding lines 368-370 (now 416-417), we acknowledge that the original wording could be misleading. Our aim was to highlight the improved plasma half-life of EPO-QMP compared to EPO-WT and EPO-AQ, emphasizing the contribution of the albumin-FcRn interaction on the PK properties of these fusion designs. We have revised the sentence for clarity, removing the comparison to darbepoetin alpha (lines 416-420):

"Nevertheless, the pharmacokinetic profile of EPO-QMP is clearly improved compared with EPO-WT in the presence of competition. On the other side, EPO-AQ was rapidly cleared in the human FcRn expressing strains regardless of mouse albumin being present or not, which is as expected and in line with a previous report [19]."

Nevertheless, we would like to emphasise that fusion to human albumin, and in particular QMP albumin with improved ability to engage human FcRn, could give proteins a prolonged plasma half-life (compared to that of the naïve recombinant protein alone), as exemplified in this paper for EPO, and for other fusion partners in previous studies by our research group (5–12). That said, we do agree with the reviewer that a major therapeutic advantage of EPO-QMP is its ability to be delivered non-invasively through the respiratory tract.

Minor concerns

3) l. 39 "should" is too strong. Please consider "could/might".

As suggested by the reviewer, we have now rephrased the sentence using "could" (*page 2, line 36*):

"Thus, long-acting EPO-QMP could be an attractive design to explore for both invasive and non-invasive delivery to anemic patients."

4) l. 56. Please add references for other long-lasting ESAs (see 2)).

The following references for other long-lasting ESAs are now added to the revised manuscript:

6. Deicher & Hörl, *Drugs* (2004)
9. Macdougall et al., *Clin. J. Am. Soc. Nephrol.* (2006)
10. Macdougall & Eckardt, *Lancet* (2006)
11. Ohashi et al., *Int. J. Nephrol. Renov. Dis.* (2012)

These are included in the following parts:

Page 3, lines 53-59:

"Another example is methoxy polyethylene glycol (PEG)-epoetin beta (Mircera, Roche) that was designed by chemically conjugating a large PEG moiety (> 30 kDa) site-specifically to recombinant EPO, resulting in a plasma half-life of 134 hours after intravenous administration in anemic patients with chronic kidney disease on dialysis [9–11]. An alternative approach could be to fuse EPO to a protein carrier that can provide prolonged plasma half-life and transcellular transport properties."

Page 16, lines 348-358:

"Recombinant human EPO exhibits a short plasma half-life of 4-11 hours after intravenous administration in humans [6], which has motivated engineering strategies to extend its systemic exposure, such as to reduce plasma clearance through increased molecular mass by introduction of N-glycan sites as for darbepoetin alfa [2,8] or by chemical conjugation to PEG as for methoxy PEG-epoetin beta [9] , respectively. Upon intravenous administration, these strategies prolong their plasma half-lives by approximately 3-fold (25.3 hours) for darbepoetin alfa and 14-fold (134 hours) for methoxy PEG-epoetin beta compared to unmodified recombinant human EPO, allowing for reduced dosing frequency [8,9,11].

An alternative approach to prolong the plasma half-life of recombinant EPO is to genetically fuse it to an FcRn ligand, which will allow receptor-mediated rescue from lysosomal degradation."

5) l. 165. What is a soluble form of the FcRn receptor?

The soluble form of FcRn refers to a recombinant truncated version of the receptor containing the three extracellular domains of the heavy chain that pairs up with the soluble β2-microglobulin (β2m) subunit. As such, the transmembrane part and cytoplasmic tail are excluded. A brief clarification has been added to the manuscript (page 8, line 165-166) and to the Methods section (page 23, lines 491-492):

Page 8, line 165-166:

"…where a recombinant truncated soluble form of the receptor, containing its extracellular domains,…"

Page 22, lines 491-492:

"A baculovirus expression vector system was used to produce soluble truncated his-tagged human FcRn containing its extracellular domains [40-42]."

6) l. 482-483. Please provide limits of quantification for EPO ELISAs. Include these limits in Fig. 4.

We have defined the lower limit of quantification (LLOQ) for each ELISA as the lowest value Prism can interpolate using the respective standard curves. Notably, as the samples are diluted in assay buffer, the LLOQ for each sample must be multiplied by the dilution factor used. This varies between assays, and also within an assay, as the more concentrated samples are diluted more in order to give OD signal within the exponential phase of the respective standard curve. Moreover, we have defined a lower limit of detection (LLOD) for each ELISA experiment as the mean OD value from blank wells + 10 × SD of these blanks. Where the interpolated unknown values fall below the LLOD, they were excluded from presentation and analyses. This is now described in the figure legend of Figure 4 (highlighted in yellow):

"Plasma concentrations were quantified using a two-way anti-albumin ELISA and the graphs show all values interpolated using Prism, where values below lower limit of detection (LLOD) and those Prism cannot interpolate were defined as not detected (ND)."

A description of the definition of LLOD, LLOQ and of data presentation is added to the ELISA section in the Methods (page 25, line 567-572):

"Data points presented were above the lower limit of detection (LLOD) and above the lower limit of quantification (LLOQ). The LLOD for each ELISA run was defined as the mean OD value of blank wells

(or those containing naïve serum) + 10 × SD of these, and the LLOQ was defined as the values by which Prism manages to interpolate using the respective standard curves. In cases where the LLOD was above the LLOQ, the values were excluded from the figure presentation and the NCA.“

7) l. 620. Why did blood sampling not last until day 30, as for IV delivery?

For the measurement of erythrocyte and hematocrit counts after intranasal delivery of EPO (previous line 620, now Methods lines 730-731 on page 31, and Figure 6f–g), we continuously analyzed whole blood immediately after sampling. We observed a progressive reduction in erythrocyte counts and hematocrit values for more than a week leading up to day 21, at which point the values for the mice treated with EPO-QMP, darbepoetin alfa, or naïve controls had only neglectable differences, and there were no longer a significant difference between EPO-QMP and the naïve controls (see Supplementary Table 3). As further sampling beyond day 21 was unlikely to yield additional data, the mice were euthanized at this time point to reduce unnecessary stress and handling, in accordance with the 3R principle of refinement.

8) l. 630. Normalizing EPO PK profiles by day 1 measurement complicates analysis. Please consider using actual data in Fig. 4 as in Supplementary Figure 4 C but in semi-log scale (see 1)).

We thank the reviewer for addressing this aspect. Figure 4h and j in the original manuscript displayed plasma concentration per day compared to day 1, which was the basis for the calculation of beta phase plasma half-life values performed in Excel. During the revision process, we have decided to report on the plasma half-life values derived from the gPKPDSim application in MatLab (please see Major Considerations comment 1 above). However, comparing elimination of test articles from day 1 is still relevant as it shows elimination after the initial albumin fusion biodistribution phase, which is important when determining the effect FcRn-ligand competition has on the various albumin designs. Nonetheless, we agree that illustrating actual plasma concentrations is highly important, especially in regard to evaluating target exposure. As such, we have modified Figure 4 to display plasma concentrations (nM) in semi-log scale and moved the clearance curve displaying percentage remaining compared to day 1 to Supplementary Figure 4c.

9) l. 637-639. Specify what data were used for NCA analysis by gPKPDsim. These data should be presented in Fig. 4 or in Supplementary Materials. Describe how gPKPDsim selects terminal phase of PK data for T1/2 calculation. State if this is consistent with Fig. 4.

The protein concentrations detected above the LLOQ and LLOD at all time points, as visualized in Figure 4h and j, were used as input for all NCA in the revised manuscript, now specified in the revised manuscript; Methods (page 30, lines 704-705) and Table 3 (page 48, lines 1028-1033). The data used for NCA is available in the Source data file.

Page 30, lines 704-705:

"…calculated using NCA including all time points above LLOD and/or LLOQ,…"

Page 48, lines 1028-1033:

"Pharmacokinetic parameters for EPO-WT, EPO-AQ, and EPO-QMP administered to Tg32 or Tg32 AlbKO mice (2 mg/kg; 23.6 nmol/kg) by either intravenous (i.v.) or subcutaneous (s.c.) injection were calculated by applying the plasma concentration-time data in Figure 4h and j, and in Supplementary

Figure 4f to the gPKPDsim application in MatLab. Data are presented as the mean ± SD (Tg32, *n* = 6; Tg32 AlbKO, *n* = 5)."

The half-life values calculated using the Excel formula in the original manuscript have been replaced with those from the NCA, to make the reporting consistent throughout the manuscript.

The SimBiology-based gPKPDsim application in MatLab uses the following formula to calculate plasma half-life:

$$T_{1/2} = \frac{\ln(2)}{\lambda_z}$$

where λ_z (the terminal rate constant) describes the slope of the terminal phase of the concentration–time curve. This equation is widely validated and commonly used in clinical pharmacokinetic practice. To estimate λ_z, SimBiology automatically evaluates several possible regressions using the last *n* data points of the terminal phase (where *n* can be 3, 4, 5, …). Concentrations are log-transformed, and the data are fitted to a linear regression. Among these fits, the algorithm chooses the regression that uses the largest number of data points with the highest adjusted R^2 (2).

10) Table 1. Please provide standard errors of estimates for Ka and Kd. Units for Ka are wrong.

We thank the reviewer for the attention towards the units of the kinetic constants presented in Table 1. The units for k_a and k_d have been corrected in the revised manuscript Table 1 and Supplementary Table 1. Moreover, the standard errors for k_a and k_d have been included in Table 1 and Supplementary Table 1, and the Method section, page 27, lines 624-629 and page 25, lines 583-589, updated accordingly:

"Binding responses from reference CM5 flow cells and blank injections were subtracted to correct for non-specific binding and bulk refractive index effects using the BIAevaluation software version 4.1 (Cytiva). Kinetic parameters were obtained by globally fitting all concentrations of each titration series, including duplicate injections, to a 1:1 Langmuir binding model, yielding a single set of association (k_a) and dissociation (k_d) rate constants per experiment with standard errors derived from the covariance matrix of the fit."

11) Table 3. Please use molar units for NCA parameters AUC, Cmax to enable comparison with binding parameters from Table 1 and HERA results from Fig. 4. Units for Dose should be ug (provide molar equivalent). All parameters should be reported as mean +/- SD of N=5 animals.

We agree that consistency in units across the manuscript is important as it allows for comparisons of different experiments. We have therefore updated the NCA parameters to molar units including SD (updated Table 3). At the same time, we would like to caution the reviewer and the readers against directly comparing these pharmacokinetic parameters with binding parameters from Table 1 or HERA results from Figure 4. While using the same units allows for a conceptual understanding of relative concentrations, the underlying measurements represent diverse biological settings. Results from HERA provide information about in vitro FcRn-mediated cellular recycling, while AUC and Cmax give insights into systemic exposure in vivo. Although both these phenomena are influenced by the binding kinetics of the protein designs to FcRn, the clearance of EPO-based proteins in vivo is also influenced by for example the accessibility of the EPOR. Therefore, any comparison should be made with these distinctions in mind.

Information about the administered dose is reported throughout the manuscript, both in Results, Figure legends, legend of Table 3 and in Methods. In the two latter, the dose is described as both mg/kg and nmol/kg or as µg with µL indicated and µM (page 29, lines 682-683 and 687, page 30, lines 712 and page 31, lines 728-729). The legend of Table 3 now specifies that all parameters are presented as mean ± SD, as well as the group sizes.

11) Fig 2. Please keep the same range for Y axes of plots in Fig. 2B. Please reverse order of X axis in Fig. 2D.

The range of the Y axis of plots in Figure 2B has been altered and now show a max of 40RU for all samples. Also, the X axis in Figure 2D have been reversed.

12) Fig. 4H and 4J. Please include full EPO concentration time courses until the limit of quantification is reached. Do not use normalize data. Use molar concentrations for comparison with other results (EPOR and FcRn binding). Thie figure should clarify what data is used for calculation terminal half-life and NCA analysis (see 1)).

We thank the reviewer for the comment regarding Figures 4H and 4J. We agree that showing concentration-time courses until the limit of quantification is reached is highly relevant and have now revised the respective subfigures to show the measured molar concentration in plasma at each time point. The graphs showing the % protein level remaining over time compared to day 1 have been moved to Supplementary Figure 4c. In addition, Figure 4h and Supplementary Figure 4c have been updated with data obtained from the repeated pharmacokinetic study, which allow the reader to follow the plasma concentration of the administered fusions for a longer period due to a lower pre-dilution of plasma in glycerol for storage and consequently a more sensitive ELISA readout.

13) Fig. 6D and 6E. Please use molar concentrations to enable comparison with other results. X label for Fig. 6E should read “intravenous”. Correct label for Fig. 6H.

We thank the reviewer for the careful observation and apologize for the earlier inconsistencies in data representation. We have now adjusted the data in Fig. 6e and i, as well as Supplementary Fig. 5b and d, to molar concentrations.

In the case of Fig. 6d and h, as well as Supplementary Fig. 5a and c, the measurements were performed using a commercial EPO ELISA kit, and for these datasets we have retained the original units (mIU/mL). The conversion of the derived values into molar concentrations is unfortunately not straightforward, as the ELISA kit standard is defined in international units (IU) and neither a defined conversion factor nor a description of the exact composition of the standard curve are provided by the manufacturer. Without this information, converting the data into molar units would introduce uncertainty and potentially misrepresentation of the results. For this reason, we have chosen to present the data in the same units as recommended by the manufacturer’s protocol, ensuring consistency with the validated assay.

We apologize for the errors in the label of the X-axes of Figure 6e and h and thank the reviewer for its attention. The labels of the X-axes of Figure 6e and h now read “Days post intravenous delivery” and “Days post intranasal delivery”, respectively.

14) Supplementary Figure 3 is too small.

We thank the reviewer for pointing this out. Supplementary Figure 3 has been prepared using the same scaling factor as the other figures in the manuscript. We acknowledge that it may appear

smaller, but adjusting the scaling of this figure independently would introduce inconsistency across the figures and have therefore chosen to keep the figure size to maintain a uniform scaling factor throughout. However, we have adjusted the x-axis maximum number from 20 RU to 16 RU.

15) Supplementary Figure 5. Please use molar concentration units

We apologize for the repeated omission of molar concentration units. We have now revised Supplementary Figure 5b and d to display the data in molar units. For Supplementary Figure 5a and 5c, we refer the reviewer to our response to Comment 13 above, explaining why conversion to molar concentrations is not feasible in those cases.

16) Supplementary Table 1. Please see 10)

The units for k_a and k_d have been corrected in the resubmitted manuscript Table 1 and Supplementary Table 1. Moreover, the standard errors for k_a and k_d have been included in Table 1 and Supplementary Table 1, and the Method section, page 27, lines 624-629 and page 25, lines 583-589.

Reviewer #2 (Remarks to the Author):

The manuscript, "A human erythropoietin-1 albumin fusion protein..." is notably well-written with excellent experimental detail provided to support the major claims of increased half-life and improved absorption of the EPO-QMP fusion protein. The authors provide insight into the specific applications of the EPO-QMP and to the utility of the approach for other fusion proteins. I have two recommendations:

1. Provide a summary of the limitations of the study, including the lack of evaluating PKPD from subcutaneous injections, which is a major route of administration for EPO and EPO-derived therapeutics.

We appreciate that subcutaneous delivery is a major route of administration for EPO and EPO-derived therapeutics, and thank the reviewer for pointing this out. Delivery through the subcutaneous route leads to a delayed max plasma level compared to intravenous administration due to gradual absorption from tissue to the blood, which can be of advantage as it often results in lower fluctuations in plasma levels.

Acknowledging the lack of a PK evaluation of the EPO-albumin fusion protein design after subcutaneous delivery as a limitation of the study, we have performed a new PK study comparing intravenous and subcutaneous delivery. This experiment, reported in revised Supplementary Figure 4d, confirms that our protein design can be delivered subcutaneously, with peak plasma levels observed after 24 hours. Moreover, delivery through the subcutaneous route retained the extended plasma half-life conferred by the QMP albumin variant observed when delivered intravenously, as the half-life values following intravenous and subcutaneous delivery were non-statistically different (5.7 ± 2.2 days versus 4.9 ± 1.3 days).

The comparison between intravenous and subcutaneous delivery has been included in the revised manuscript as follows; Results (pages 11-12, lines 250-263), Discussion (page 18, lines 400-404), Table 3, and Supplementary Figure 4e-f.

Pages 11-12, lines 250-263:

"Furthermore, we addressed how a subcutaneous delivery route would affect the plasma half-life and pharmacokinetics of EPO-QMP with the same dose as before (2.0 mg/kg). While the amounts detected after intravenous delivery decreased 1.4-fold between 8 hours and 24 hours, specifically from 111.8 to 78.4 nM, subcutaneous delivery resulted in a 1.7-fold increase, from 37.7 to 63.3 nM in the same interval (Supplementary Figure 4e). For the timepoints following 24 hours, comparable reductions in detected EPO-QMP were observed for both administration routes (Supplementary Figure 4e-f). Mice given EPO-QMP by the subcutaneous route showed a maximum plasma concentration (Cmax) of 63.3 nM, 6.7-fold lower than that measured after intravenous delivery (Table 3). In addition, the time point at which Cmax was measured was shifted, from 5 minutes with intravenous delivery to 24 hours with subcutaneous delivery (Supplementary Figure 4d-f). Despite the differences in amounts detected at early time points, the plasma half-life after subcutaneous delivery (4.9 ± 1.3 days) was not statistically different from that after intravenous delivery (Table 3, Supplementary Figure 4f, Supplementary Table 2)."

Page 18, lines 400-404:

"A common route of administration for protein-based therapeutics, including those that are EPO-based, is by subcutaneous delivery. As such, we compared intravenous and subcutaneous administration of EPO-QMP, which resulted in similar plasma elimination curves and derived plasma half-life values, supporting both delivery strategies as relevant for future studies."

2. Modified proteins have the potential to be immunogenic. Although a complete study into immunogenecity is arguably beyond the scope of this report, any guidance or relevant references in this regard would be useful.

We thank the reviewer for highlighting this important point. We note that biologics containing human albumin as a fusion partner or carrier are approved for clinical use and show favorable safety profiles. For instance, Idelvion, a recombinant fusion protein linking coagulation factor IX with WT human albumin (rIX-FP), which is extensively used to treat hemophilia B patients, did not show detectable levels of neutralizing antibodies in a phase III clinical trial (16),

While Idelvion is built on WT human albumin, our protein design is based on an engineered albumin variant (QMP) with three amino acid substitutions that enhance pH-dependent binding to human FcRn and extend its plasma half-life. To test if antibodies were raised against the EPO-albumin fusion proteins or naked QMP albumin in the Tg32 mice, we compared plasma samples collected on day 27 with that of early time points. The results showed no signs of immunogenicity towards either the fusion proteins or QMP albumin alone as presented in Supplementary Figure 4g-h.

Further, we have previously demonstrated that in vivo administration of antigen albumin fusions based on WT human albumin and the QMP variant did not elicit detectable anti-albumin antibody responses in transgenic mice expressing both human albumin and human FcRn, even in the presence of adjuvant and boosting (10). Thus, our findings support that the engineered QMP variant may be as safe as that of WT albumin. Nevertheless, we acknowledge that this must be assessed on a case-to-case basis, and a clear answer will not be revealed until a QMP-containing fusion design is tested in a first-in-human study. In this regard, the following has been added to the results (page 12, line 265-270) and discussion (page 20, lines 445-456):

Results (page 12, line 265-270):

"The potential generation of antibodies against the human EPO-albumin fusions or unfused QMP albumin in Tg32 mice was evaluated by ELISA. Specifically, we compared plasma samples collected at

early time points (5 minutes for intravenous and 8 hours for subcutaneous) with that of samples collected on day 27. The results showed no sign of immunogenicity raised towards either the EPO albumin fusions (Supplementary Figure 4g) or unfused QMP albumin (Supplementary Figure 4h)."

Discussion (page 20, lines 445-456):

"A key aspect in a translation perspective would be to determine the safety profile of the fusion design and the potential for development of anti-drug antibodies. Notably, the WT human albumin-FIX fusion Idelvion is extensively used for prophylactic treatment of patients with hemophilia B, and neutralizing antibodies against this albumin fusion were not detected in a phase 3 clinical trial [38]. Moreover, we have demonstrated that introducing the three QMP amino acid substitutions did not change the immunogenicity profile compared to that that of a WT human albumin fusion when used in design of subunit vaccines, as no antibody responses were detected against either human albumin variants after two doses of adjuvanted WT- or QMP-based vaccines in mice expressing both human FcRn and human albumin [40]. In line, antibodies against the EPO-albumin fusion or against QMP albumin were not detected in the plasma samples collected from Tg32 mice on day 27. These findings support a favorable safety profile of therapeutic fusion designs built on WT- and QMP-containing albumin."

The following references have been added to the revised manuscript to guide the reader regarding immunogenicity:

36. Aaen et al., *Nat. Commun.* (2025)
37. Nilsen et al., *Commun. Biol.* (2025)
38. Santagostino et al., *Blood* (2016)
40. Anthi et al., *Nat. Commun.* (2025)

List of references:

1. Hallare J, Gerriets V. Elimination Half-Life of Drugs. In: StatPearls [Internet]. Treasure Island (FL): StatPearls Publishing; 2025 [cited 2026 Jan 29]. Available from: http://www.ncbi.nlm.nih.gov/books/NBK554498/

2. Noncompartmental Analysis - MATLAB & Simulink [Internet]. [cited 2026 Feb 5]. Available from: https://se.mathworks.com/help/simbio/ug/non-compartmental-analysis.html

3. Egrie JC, Browne JK. Development and characterization of novel erythropoiesis stimulating protein (NESP). Br J Cancer. 2001 Apr;84(Suppl 1):3–10.

4. Jarsch M, Brandt M, Lanzendörfer M, Haselbeck A. Comparative Erythropoietin Receptor Binding Kinetics of C.E.R.A. and Epoetin-β Determined by Surface Plasmon Resonance and Competition Binding Assay. Pharmacology. 2007 Nov 1;81(1):63–9.

5. Bern M, Nilsen J, Ferrarese M, Sand KMK, Gjølberg TT, Lode HE, et al. An engineered human albumin enhances half-life and transmucosal delivery when fused to protein-based biologics. Sci Transl Med [Internet]. 2020 Oct 14 [cited 2020 Nov 25];12(565). Available from: https://stm.sciencemag.org/content/12/565/eabb0580

6. Mester S, Evers M, Meyer S, Nilsen J, Greiff V, Sandlie I, et al. Extended plasma half-life of albumin-binding domain fused human IgA upon pH-dependent albumin engagement of human FcRn in vitro and in vivo. mAbs. 2021 Jan 1;13(1):1893888.

7. Lombardi S, Aaen KH, Nilsen J, Ferrarese M, Gjølberg TT, Bernardi F, et al. Fusion of engineered albumin with factor IX Padua extends half-life and improves coagulant activity. Br J Haematol. 2021;194(2):453–62.

8. Benjakul S, Anthi AK, Kolderup A, Vaysburd M, Lode HE, Mallery D, et al. A pan-SARS-CoV-2-specific soluble angiotensin-converting enzyme 2-albumin fusion engineered for enhanced plasma half-life and needle-free mucosal delivery. PNAS Nexus. 2023 Dec 1;2(12):pgad403.

9. Mester S, Chan C, Lustig M, Foss S, Jansen JHM, Leangen Herigstad M, et al. Engineering of anticancer human immunoglobulin A equipped with albumin for enhanced plasma half-life. PNAS Nexus. 2025 Feb 11;4(2):pgaf042.

10. Anthi AK, Kolderup A, Vaage EB, Bern M, Benjakul S, Tjärnhage E, et al. An intranasal subunit vaccine induces protective systemic and mucosal antibody immunity against respiratory viruses in mouse models. Nat Commun. 2025 May 1;16(1):3999.

11. Aaen KH, Testa MF, Nilsen J, Tarantino R, Canepari C, Benedusi M, et al. Tailored collagen binding of albumin-fused hyperactive coagulation factor IX dictates in vivo distribution and functional properties. Nat Commun. 2025 Sep 29;16(1):8433.

12. Nilsen J, Aaen KH, Benjakul S, Ruso-Julve F, Greiner TU, Bejan D, et al. Enhanced plasma half-life and efficacy of engineered human albumin-fused GLP-1 despite enzymatic cleavage of its C-terminal end. Commun Biol. 2025 May 26;8(1):1–17.

13. Salmonson T, Danielson B, Wikstrom B. The pharmacokinetics of recombinant human erythropoietin after intravenous and subcutaneous administration to healthy subjects. Br J Clin Pharmacol. 1990 Jun;29(6):709–13.

14. Jelkmann W. The enigma of the metabolic fate of circulating erythropoietin (Epo) in view of the pharmacokinetics of the recombinant drugs rhEpo and NESP. Eur J Haematol. 2002;69(5–6):265–74.

15. Yan X, Krzyzanski W. Quantitative assessment of minimal effective concentration of erythropoiesis-stimulating agents. CPT Pharmacomet Syst Pharmacol. 2013 Aug;2(8):1–10.

16. Santagostino E, Martinowitz U, Lissitchkov T, Pan-Petesch B, Hanabusa H, Oldenburg J, et al. Long-acting recombinant coagulation factor IX albumin fusion protein (rIX-FP) in hemophilia B: results of a phase 3 trial. Blood. 2016 Apr 7;127(14):1761–9.

REVIEWERS' COMMENTS

Reviewer #1 (Remarks to the Author):

Thank you for addressing my concerns. I do not have any further questions. One suggestion
1) Since you have measured plasma concentrations for i.v. and s.c. administration it will be helpful to report bioavailability either in Table 3 or in Supplementary Figure 4.

We thank the reviewer for the suggestion to report bioavailability for the SC administration. This has been added to Table 3, with the result of 76%.

Reviewer #2 (Remarks to the Author):

The authors have made substantial, thoughtful changes to respond to previous reviewers' concerns. While these are mostly sufficient, there are two issues that could be addressed to improve the manuscript further:

1. The authors acknowledge they still cannot define a true terminal half-life at physiologically relevant EPO concentrations (10-100 pM), yet the manuscript continues to report "plasma half-life". The authors report that when they attempted dosing at 2 µg/kg (to get into the 10-100 pM) the fusion protein could not be detected above background at any timepoint. In addition, there is a new statement noting that "the plasma elimination curve for EPO-QMP in the Tg32 mice display several phases, indicating multiple modes of clearance mechanisms"; however, it is not entirely clear if the latter phase reflects a EPOR-mediated elimination only (or other non-specific clearance only), or if there is an additional terminal phase at physiologic relevant concentrations below 100 pM. Although this limitation is honestly disclosed, the half-life differences are central to the conclusions of the manuscript and authors should carefully consider revising their claim of "plasma half-life" to "apparent terminal half-life" or something else more transparent.

We thank the reviewer for this valid comment. We suggest retaining the term "plasma half-life", as we believe it appropriately encompasses both EPOR-mediated clearance and any other clearance mechanisms that may occur simultaneously. Since we are unable to distinguish the relative contributions of the individual clearance pathways in detail, we consider this broader term to be the most appropriate. This approach also reflects our intention to avoid misusing the term "terminal half-life" or implying that the observed pharmacokinetics are solely determined by EPOR-mediated clearance, as there remains uncertainty regarding the contribution of the different clearance mechanisms.

2. The justification for using DARB as a reference is better explained, but somewhat side-steps the major concern that the comparison to DARB only shows that modification of EPO with QMP improves half-life over an unmodified form of EPO, but does not demonstrate equivalence, or superiority, over other half-life-extending forms of EPO.

We appreciate the reviewer's comment and would like to further clarify our rationale for selecting darbepoetin alfa as the comparator throughout the study. Our intention

was to use a single clinically approved ESA as a consistent reference across all relevant experiments.

Although methoxy polyethylene glycol-epoetin beta has a longer plasma half-life in humans than darbepoetin alfa, it also exhibits lower affinity for the human EPOR. In addition, both approved ESAs are engineered molecules: darbepoetin alfa contains amino acid substitutions that increase glycosylation, whereas methoxy polyethylene glycol-epoetin beta is modified by conjugation to a large PEG moiety. Consequently, neither represents an unmodified EPO molecule.

Our objective was not simply to compare plasma half-life, but rather to evaluate the overall pharmacological profile of EPO-QMP, including receptor binding, biological activity and pharmacokinetic properties. We therefore considered darbepoetin alfa to provide an appropriate balance between receptor affinity and prolonged systemic exposure, making it a suitable single benchmark for the study.

We acknowledge that inclusion of additional comparators, such as methoxy polyethylene glycol-epoetin beta and an unmodified ESA (e.g. epoetin zeta), could have provided further context. However, this was beyond the scope of the present work, and we chose to maintain a single clinically relevant reference to ensure consistency across the different experimental analyses.

To explicitly acknowledge this limitation, we added the following statement to the manuscript during the previous round of revision:

"Of note, a crucial aspect for efficacy is systemic exposure over time and thus the plasma half-life. Further studies are needed to compare the efficacy of EPO-QMP with other ESAs."

We believe this statement appropriately recognizes that, particularly for stimulation of erythropoiesis, prolonged systemic exposure may be at least as important as receptor affinity, and that additional comparative studies with other approved ESAs would be valuable in future work.
